# Association of nicotine dependence and gut microbiota: a bidirectional two-sample Mendelian randomization study

**DOI:** 10.3389/fimmu.2023.1244272

**Published:** 2023-11-07

**Authors:** Yuexuan Chen, Mengjiao Zhao, Kaisong Ji, Jingjing Li, Shuxin Wang, Liming Lu, Zhenhu Chen, Jingchun Zeng

**Affiliations:** ^1^ The First Clinical College, Guangzhou University of Chinese Medicine, Guangzhou, China; ^2^ South China Research Center for Acupuncture and Moxibustion, Medical College of Acu-Moxi and Rehabilitation, Guangzhou University of Chinese Medicine, Guangzhou, China; ^3^ Department of Acupuncture, Baoan District Hospital of Traditional Chinese Medicine, Shenzhen, China; ^4^ Department of Acupuncture, The First Affiliated Hospital of Guangzhou University of Chinese Medicine, Guangzhou, China

**Keywords:** gut microbiota, Mendelian randomization, nicotine dependence, causality, genetics

## Abstract

**Background:**

Nicotine dependence is a key factor influencing the diversity of gut microbiota, and targeting gut microbiota may become a new approach for the prevention and treatment of nicotine dependence. However, the causal relationship between the two is still unclear. This study aims to investigate the causal relationship between nicotine dependence and gut microbiota.

**Methods:**

A two-sample bidirectional Mendelian randomization (MR) study was conducted using the largest existing gut microbiota and nicotine dependence genome-wide association studies (GWAS). Causal relationships between genetically predicted nicotine dependence and gut microbiota abundance were examined using inverse variance weighted, MR-Egger, weighted median, simple mode, weighted mode, and MR-PRESSO approaches. Cochrane’s Q test, MR-Egger intercept test, and leave-one-out analysis were performed as sensitivity analyses to assess the robustness of the results. Multivariable Mendelian randomization analysis was also conducted to eliminate the interference of smoking-related phenotypes. Reverse Mendelian randomization analysis was then performed to determine the causal relationship between genetically predicted gut microbiota abundance and nicotine dependence.

**Results:**

Genetically predicted nicotine dependence had a causal effect on *Christensenellaceae* (β: -0.52, 95% CI: -0.934–0.106, P = 0.014). The *Eubacterium xylanophilum* group (OR: 1.106, 95% CI: 1.004-1.218), *Lachnoclostridium* (OR: 1.118, 95% CI: 1.001-1.249) and *Holdemania* (OR: 1.08, 95% CI: 1.001-1.167) were risk factors for nicotine dependence. *Peptostreptococcaceae* (OR: 0.905, 95% CI: 0.837-0.977), *Desulfovibrio* (OR: 0.014, 95% CI: 0.819-0.977), *Dorea* (OR: 0.841, 95% CI. 0.731-0.968), *Faecalibacterium* (OR: 0.831, 95% CI: 0.735-0.939) and *Sutterella* (OR: 0.838, 95% CI: 0.739-0.951) were protective factor for nicotine dependence. The sensitivity analysis showed consistent results.

**Conclusion:**

The Mendelian randomization study confirmed the causal link between genetically predicted risk of nicotine dependence and genetically predicted abundance of gut microbiota. Gut microbiota may serve as a biomarker and offer insights for addressing nicotine dependence.

## Introduction

The use of tobacco products is responsible for the deaths of nearly 8 million people worldwide each year (1) and is a significant contributor to lung cancer and cardiovascular disease (2). Although many people are aware of the detrimental effects associated with smoking, the presence of nicotine, a highly addictive substance in tobacco products makes it difficult for individuals to quit (3, 4). Nicotine is known to reinforce smoking and tobacco use behaviors that establish and sustain nicotine dependence. The majority of smokers require some form of assistance to quit, as only approximately 4% of smokers are able to quit on their own successfully (1). Nicotine dependence often presents with physical manifestations, including an increase in tolerance, withdrawal symptoms, and reduced ability to control behavior.

Nicotine is quickly absorbed via the oral mucosa and respiratory tract (5), thereby increasing the risk of related cardiovascular, respiratory, and gastrointestinal diseases (6). Smoking also increases the likelihood of developing gastrointestinal diseases, notably inflammatory bowel disease (7), irritable bowel syndrome (8), peptic ulcer disease (9), and gastrointestinal cancer (10). In addition, smoking heightens the risk of gastrointestinal infections (11), including Helicobacter pylori (12).

Gut microbiota comprises a diverse array of microorganisms that colonize the mammalian gut, including bacteria, fungi, archaea, viruses, and parasites (13). The two-way communication between gut microbes and their hosts may influence many immunity- and metabolism-related biological systems, thereby impacting host health (14). Enhancing host immunity is an important function of the gut microbiota (15). The gut microbiota competes for limited nutrients and regulates host immunity to suppress the colonization of exogenous pathogenic microorganisms (16–18). Additionally, the effects of these immune reactions can extend to almost all parts of the human body (15). When there is an imbalance in the gut microbiota and impaired intestinal barrier function, an increase in harmful pathogenic microorganisms may further induce the occurrence and development of diseases, such as hypertension (19), autoimmune hepatitis (20), cancer (21), and others.

Smoking can modify the microbiome in several regions (22), including the periodontal, intestinal, and respiratory tracts, and augments the mechanisms whereby changes in mucosal immune responses, fluctuations in intestinal cytokine levels, alteration in intestinal permeability, and epigenetic modification alter gene expression (23, 24). Prebiotics are undigestible food elements that can selectively promote the growth and function of the colonic microbiota, ultimately improving host health (25). Supplementation of probiotics and the reconstruction of a healthy microbiota in the gut are now considered effective strategies for treating diseases caused by gut microbiota dysbiosis (26–28). Therefore, using appropriate prebiotics to target specific microbial communities may be an effective approach for preventing and treating nicotine dependence. However, the causal relationship and mechanisms between gut microbiota and nicotine dependence are still unclear, which poses obstacles to the prevention and treatment of nicotine dependence. Thus, it is imperative to study the causal link between the gut microbiota and nicotine dependence.

The Mendelian randomization (MR) method is an epidemiological technique (29) that employs genetic variation as an instrumental variable to explore the putative causal effects of exposure on the onset of disease. Building upon the recent large-scale genome-wide association studies (GWAS) on the gut microbiota (30–32) and disease, we employed the Mendelian randomization approach to investigate the causal link between the gut microbiota and the risk of nicotine dependence in this study. This study aims to explore the impact of genetic prediction of nicotine dependence on the gut microbiota, and elucidate the role of the gut microbiota in the pathogenesis of nicotine dependence through genetic prediction. Furthermore, it aims to uncover the potential of genetic prediction of the gut microbiota to aid in the development of novel preventive strategies.

## Methods

### Study design

**Figure 1:**
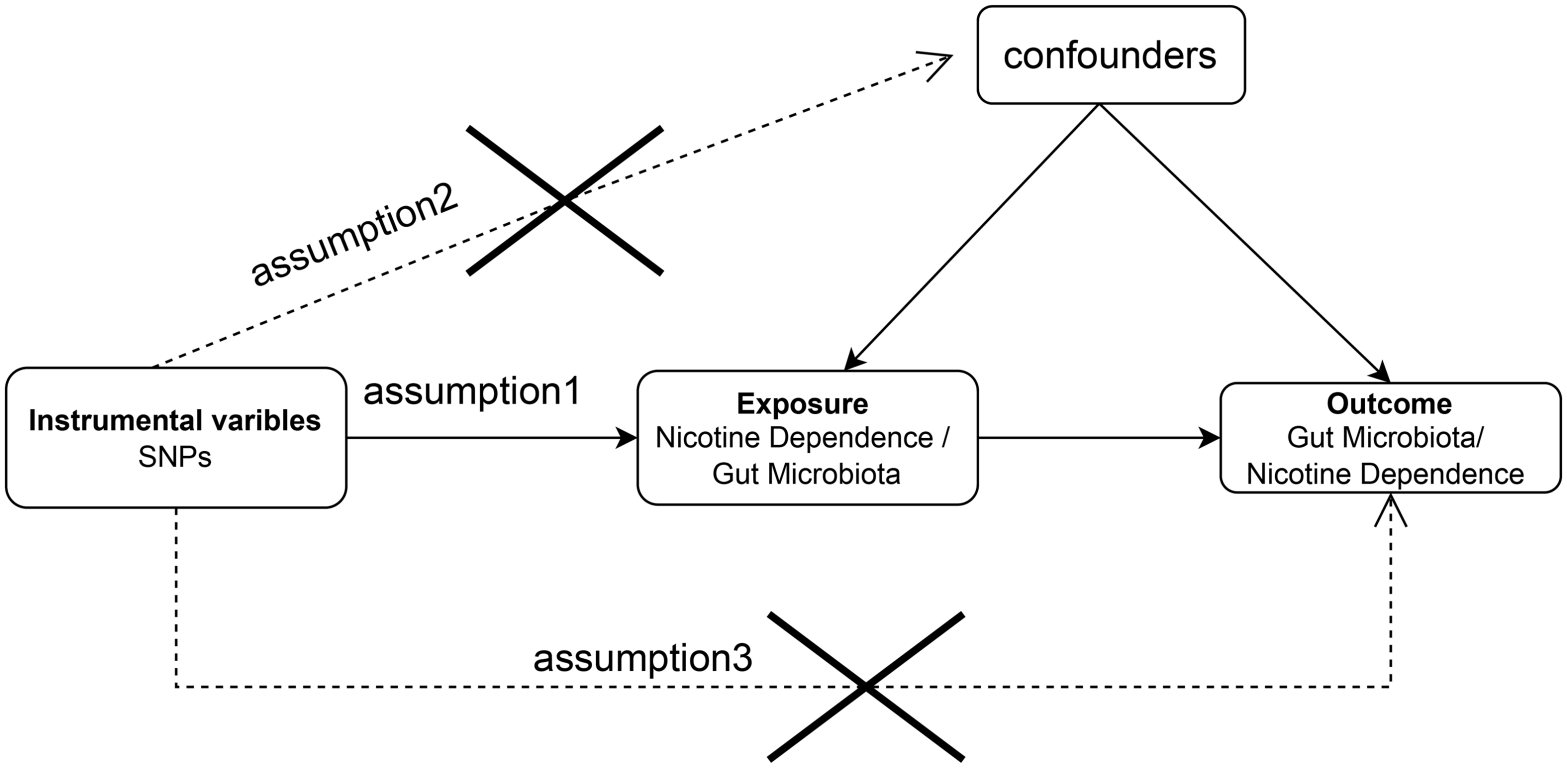
Bidirectional two-sample Mendelian randomization between nicotine dependence and gut microbiota abundance outcomes. Directed acyclic graph (DAG) of the causal relationship between nicotine dependence and gut microbiota abundance.

**Figure 2:**
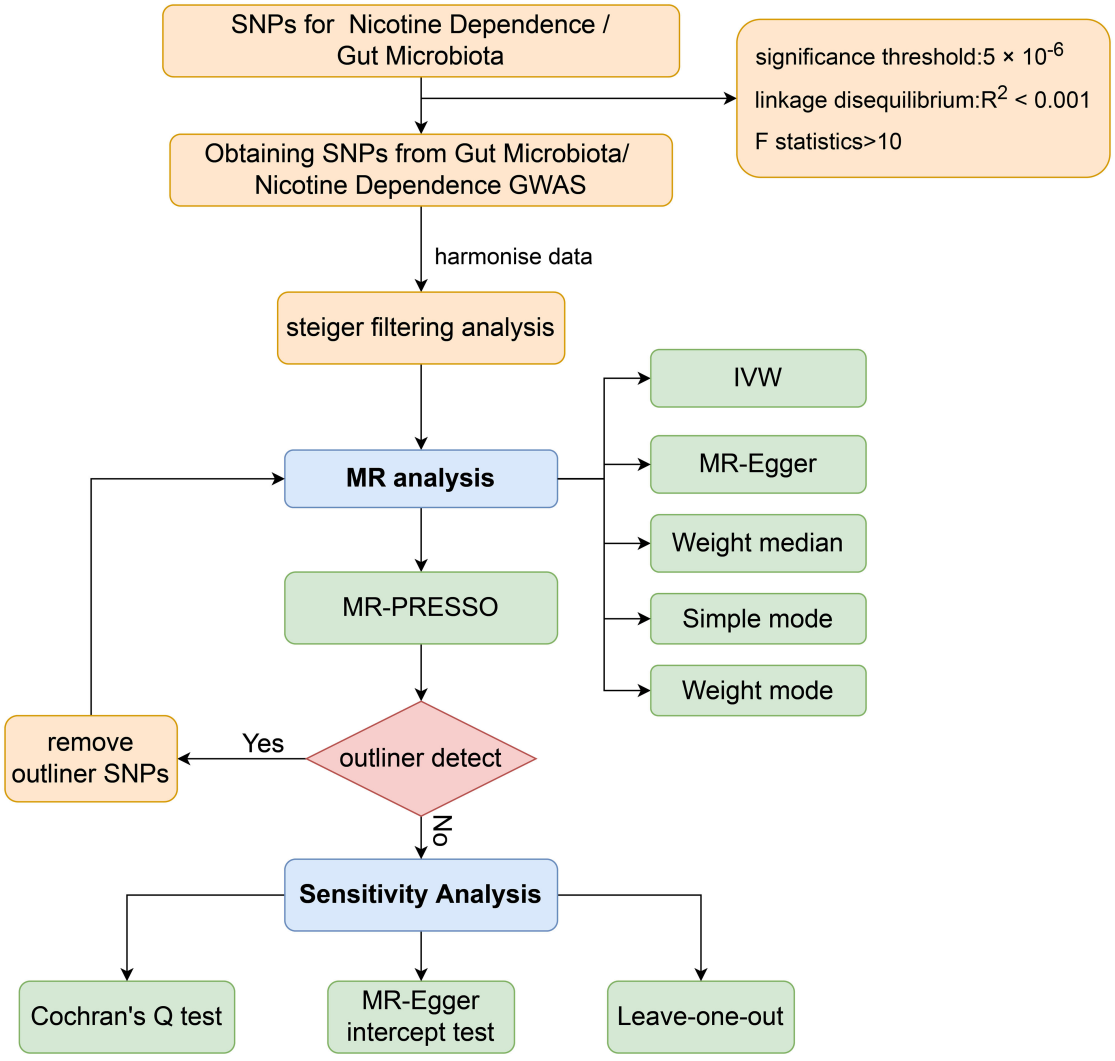
Workflow of Mendelian randomization study revealing causality between gut microbiota abundance and risk of nicotine dependence. SNP, single-nucleotide polymorphism; IVW, inverse variance weighted; MR, Mendelian randomization; MR-PRESSO, MR Pleiotropy Residual Sum and Outlier.

The aim of this study was to evaluate the causal relationship between genetically predicted nicotine dependence risk and genetically predicted abundance of gut microbiota using a Mendelian randomization method. The Mendelian randomization design consisted of three components. Firstly, the selection of genetic variants as instrumental variables for nicotine dependence. Secondly, the acquisition of a summary dataset for genetic instruments derived from a genome-wide association study of nicotine dependence, and finally, obtaining a summary dataset for single nucleotide polymorphism results. These results were used to investigate the impact of GWAS genetic instruments on gut microbiota. **Figure 1** outlines the design of the Mendelian randomization study, while **Figure 2** presents an overview of the investigation along with a flow chart. The research design of this study follows the reporting guidelines of STROBE-MR (33), and the supplementary files include the checklist based on STROBE-MR and the checklist based on Critical Appraisal Checklist for evaluating Mendelian randomization studies (34). The checklist is elaborated in detail in the supplementary materials.

### GWAS summary data for nicotine dependence

This study used the genome-wide association meta-analysis data from Hancock DB et al. to investigate nicotine dependence risk (35). The authors conducted a genome-wide meta-analysis on 38,602 former smokers of European and African American descent with mild (N = 17,796; 46.1%), moderate (N = 13,527; 35%), or severe (N = 7,279; 18.9%) nicotine dependence. Genotyping was performed on various genome-wide platforms, and after quality control, 1000G genomic interpolation was used to analyze the genotype data. Linear regression was carried out on the data and adjusted for age, sex, pedigree principal components, and cohort-specific covariates. The Genome-wide association study (GWAS) results were combined using METAL with fixed-effects inverse variance weighting meta-analysis, across all studies with FTND data to maximize statistical power. More than 99% of the former smokers were over 18 years old, and in case of the presence of relatives, family structure was adjusted. Additional information can be found in **Table S1**.

### GWAS summary data for gut microbiota

Data on the composition of human gut microbiota were obtained from the MiBioGen consortium through a large-scale multi-ethnic GWAS study (36). This study involved 18,340 participants from 24 cohorts in countries such as the United States, Canada, Israel, Korea, Germany, Denmark, the Netherlands, Belgium, Sweden, Finland, and the UK. The participants’ 16S ribosomal RNA gene sequences and genotyped data were analyzed to investigate the relationship between human autosomal genetic variation and gut microbial communities. The study included 211 taxa comprising 35 families, 20 orders, 16 phyla, 9 orders, and 131 genera.

### GWAS summary data for smoking-related phenotypes

The GWAS data for smoking-related phenotypes were obtained from a meta-analysis of GWAS summary association data from 1,232,091 individuals predominantly of European ancestry (37). The smoking phenotypes included age of smoking initiation, smoking initiation, cigarettes per day, and smoking cessation. The authors applied extensive genetic quality control and filtering to the summary statistics provided by each cohort. Imputed variants with an imputation quality below 0.3 (estimated squared correlation between imputed and true dosage) were subsequently removed. Then, the allele labels and allele frequencies of each study were compared with those of the imputation reference panel, and discrepancies were either removed or harmonized. Finally, a meta-analysis was conducted using the software package rareGWAMA based on a fixed-effect model.

### Selection of instrumental variables

This study aimed to explore the causal relationship between nicotine dependence and gut microbiota through the Mendelian Randomization analysis of instrumental variables. First, single nucleotide polymorphisms (SNPs) with a genome-wide significance threshold (5 × 10^-8^) were selected as instrumental variables (IVs) relating to nicotine dependence. After linkage disequilibrium analysis (R2 < 0.001, clumping distance = 10,000 kb), only one SNP was retained. To ensure a satisfactory number of IVs, a significance threshold of 5 × 10^-6^ for SNP versus nicotine-dependent phenotypes and a minor allele frequency (MAF) threshold of 0.01 were set. Additional linkage disequilibrium analysis (R2 < 0.001, clumping distance = 10,000 kb) was conducted on the European 1000 Genomes Project data to screen out instrumental variables that could cause biased results.

To assess the potential causal influence of gut microbiota on nicotine dependence, we analyzed genome-wide association data of gut microbes at five taxa levels: order, class, family, genus, and phylum, defining each taxon as a trait. We implemented quality control steps to select the most suitable instrument and ensure the reliability and accuracy of conclusions regarding the causal relationship between the gut microbiome and nicotine risk. Firstly, we selected snps with significance below the genome-wide statistical threshold (5 × 10^-8^), but this provided few eligible IVs. Therefore, we lowered the threshold to P<5×10^-6^, which is more comprehensive. We then used a MAF threshold of 0.01 for variants of interest and performed an LD analysis (R2 < 0.001, with a clumping distance of 10,000 kb) to evaluate LD among the included snps.

We evaluated the strength of instrumental variables by computing the F-statistic as F = R2 × (N - 2)/(1 - R2), where R2 represents the proportion of variation in the exposure factor clarified by each instrumental variable while N represents the sample size for the GWAS that relates to the exposure (38). In turn, R2 is calculated as (2 x EAF x (1 - EAF) x beta^2^)/[(2 x EAF x (1 - EAF) x beta^2^) + (2 x EAF x (1 - EAF) x N x SE(beta)^2^)], where EAF is the effect allele frequency, beta is the estimated genetic effect of the exposure factor, N is the GWAS sample size for the SNP-exposure correlation, and SE (beta) refers to the genetic effect’s standard error (39). Instrumental variables having a F-statistic <10 for weak instruments may suggest a possible bias and need to be removed. Meanwhile, those having a F-statistic >10 are included for further analysis.

Steiger filtering analysis (40) was further used to determine the directional effect of individual instrumental variable SNPs on the outcome. A “TRUE” result predicts the expected direction of association. SNPs that are shown as “False” in the Steiger filtering analysis will be excluded and not included in the subsequent Mendelian randomization analysis.

### Statistics analysis

Statistical analyses were performed using R software version 4.2.2, utilizing the R packages “TwoSampleMR” (v.0.5.6) (40), “MRPRESSO” (v.1.0) (41), and “MendelianRandomization” (42) (v.0.7.0) in order to carry out a Mendelian randomization (MR) analysis on the causal relationship between nicotine dependence and gut microbiota. Statistical significance was determined at p<0.05 to establish causality.

A multiple test significance threshold was set at 0.05/n (where n represents the number of independent bacterial taxa at the corresponding taxonomic level) due to the numerous comparisons that took place at each character level, such as phylum, class, order, family, and genus. Significance values that fell between the multiple test significance threshold and 0.05 were considered potentially significant.

### Two-sample Mendelian randomization

The primary analysis used inverse variance-weighted (IVW) to explore the potential causal relationship between gut microbiota abundance and nicotine dependence. The IVW method is widely applied in Mendelian randomization studies and provides reliable causal estimates in the absence of horizontal pleiotropy. IVW method, namely the meta-analysis of the variant-specific Wald ratios of each variant (i.e., the beta coefficient of the exposure SNP divided by the beta coefficient of the outcome SNP) (43), is used to provide a combined estimate of the causal estimates for each SNP in each potential direction of effect. The IVW method assumes independence of genetic variation and serves as an effective tool for instrumental variable analysis. However, it may ignore the mediating effects of other risk factors or potential pleiotropy, and bias may occur when there is horizontal pleiotropy between instrumental SNPs (44). In addition to the exposure, it may also affect the outcome of interest through causal pathways, resulting in a violation of the instrumental variable assumption of Mendelian randomization. Therefore, we additionally applied the methods of weighted median, MR-Egger, simple mode, and weighted mode. Based on the assumption of Instrument strength independent of direct effect (InSIDE), the MR-Egger regression method conducts weighted linear regression to generate consistent causal effect estimates independent of IV effectiveness (44). However, the MR-Egger regression method has relatively poor accuracy and is susceptible to the influence of peripheral genetic variation (45). The weighted median method can achieve unbiased estimation of the effect, which does not rely on the InSIDE assumption and thus holds significant advantages over the MR-Egger regression method (46). Specifically, it is an excellent alternative method that allows stable estimation of the causal effect when the weight of the causal effect calculated by effective instrumental variables exceeds 50%, while providing lower Type I error. Finally, the weighted mode method was employed to assess the overall causal effect of a large number of genetic instruments. In many cases, this method yields lower Type I error, less bias, and lower computational complexity compared to the primary methods (47).

### Sensitivity analysis

We conducted several sensitivity analyses consisting of tests such as Cochran’s Q statistic, funnel plots, leave-one-out analysis, and the MR-Egger intercept test. Cochran’s Q test revealed heterogeneity in the instrumental variables in case the p-value was lower than 0.05. The “leave-one-out” method was applied to validate the causal relationship between nicotine dependency and gut microbiota abundance. The fluctuations observed in results before and after SNP removal demonstrate the stability of the causal association between the exposure variable and the outcome. In the MR-Egger intercept test, a non-zero intercept reflects the presence of directional pleiotropy and represents the mean pleiotropic effect of genetic variation (44).

For detecting and correcting pleiotropic outliers, we employed the mendelian randomized pleiotropic residuals and outliers (MR-PRESSO) method (48). The method tested for overall heterogeneity through regressing SNP-outcome associations on SNP-exposure associations. The observed distance of each SNP from the regression was then matched with the expected distance under the original hypothesis of no pleiotropy. Upon detecting outliers in the MR-PRESSO analysis, we removed them and repeated the Mendelian randomization analysis mentioned above.

### Multivariable Mendelian randomization

**Figure 3:**
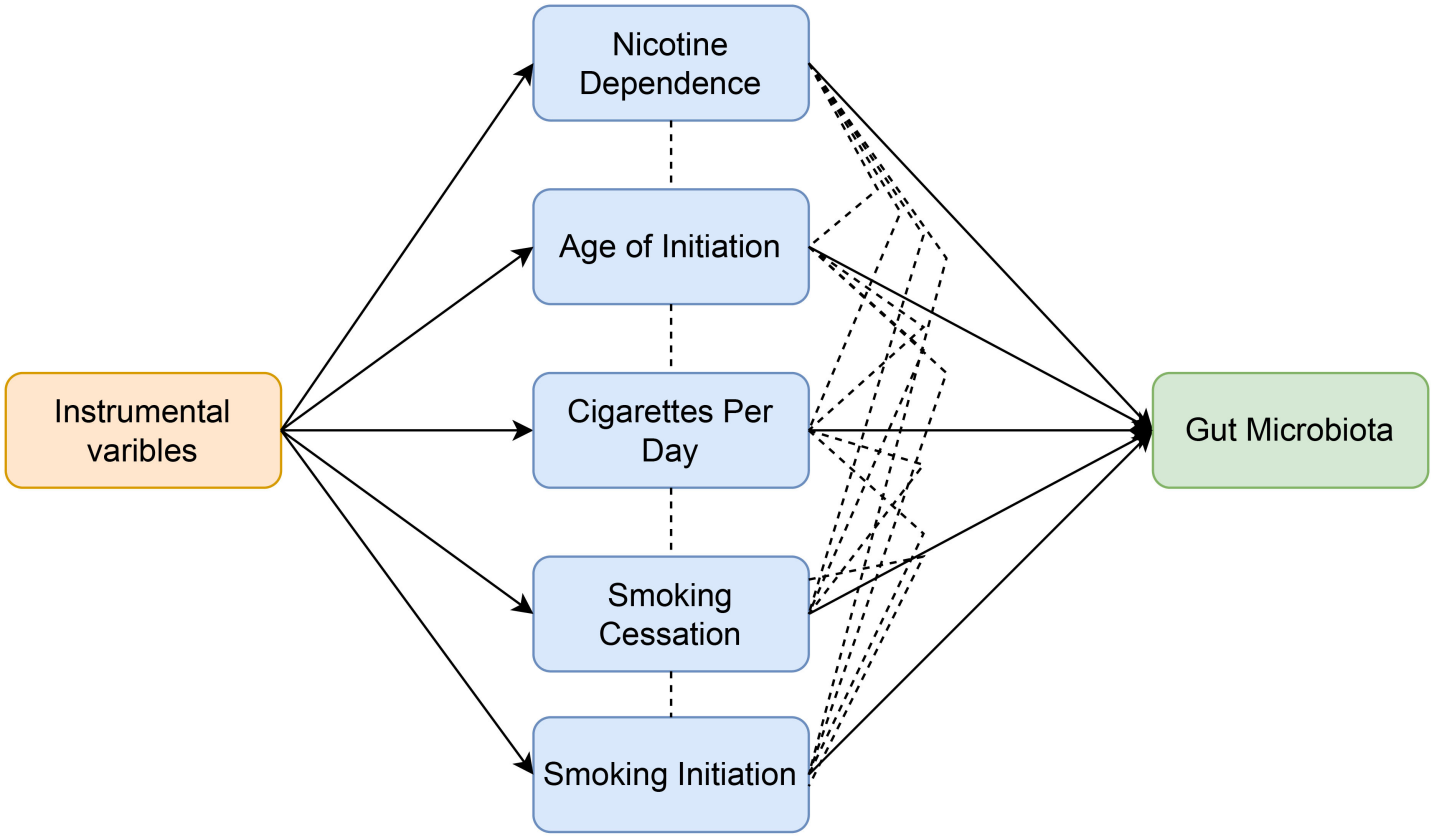
Workflow of multivariable Mendelian randomization study revealing causal effect of nicotine dependence on gut microbiota abundance by evaluating the moderating effect of smoking-related phenotypes.

In order to evaluate the moderating effect of smoking-related phenotypes on the causal relationship between nicotine dependence and gut microbiota, SNPs related to smoking-related phenotypes and nicotine dependence were extracted and selected as instrumental variables (IVs) for Multivariable Mendelian randomization (**Figure 3**). The GWAS p-value threshold between SNPs and phenotypes was set at 5 × 10^−6^. A block window of 10,000 kb and r2 = 0.001 were chosen to remove linkage disequilibrium. Cross-instrumental variables were harmonized with the outcome to obtain adjusted assessments for causal effects. A multivariable random-effect IVW model and MR-egger model were constructed in Multivariable Mendelian randomization. Statistical significance was determined at p<0.05 to establish causal relationships.

### Reverse Mendelian randomization analysis

To investigate whether gut microbiota abundance is associated with the risk of nicotine dependence, we conducted a reverse Mendelian randomization (MR) analysis using SNPs related to gut microbiota abundance as instrumental variables (where gut microbiota is the exposure and nicotine dependence risk is the outcome).

### Standard protocol approval, registration and patient consent

The GWAS data used in this study were all from publicly available databases. The summary statistics of nicotine dependence, smoking-related phenotypes and gut microbiota abundance do not contain any personal information, and each GWAS has received ethical approval from the relevant ethical review board.

## Results

### Causal effect of nicotine dependence on gut microbiota

17 SNPs met the instrumental variable screening criteria for nicotine dependence, and all had an F-statistic >10 indicating no weak instrumental bias (**Table S2**). The F-statistic for the instrumental variable lies between 20.91 and 62.95. In addition, Steiger filtering analysis helped to exclude SNPs with reverse causal directions (three from genus Victivallis and one from genus Prevotella9) (**Table S3**). As the summary data for SNP results were not extracted for the genera *Erysipelotrichaceae UCG003*, *Lachnospira* and *Blautia*, a mendelian randomization analysis was carried out for the outcomes, including a combined total of 208 gut microbiota classifications to investigate the relationship between nicotine dependence and gut microbiota (**Table S4**), namely 16 classes, 128 genera, 35 families, 20 orders, and 9 phyla.

The significance thresholds for multiple comparisons at different taxon levels were set at: phylum (p = 5.560×10^-3^), class (p = 3.125×10^-3^), order (p = 2.500×10^-3^), family (p = 1.429×10^-3^), and genus (p = 3.906×10^-4^), with adjusted P-values based on Bonferroni correction.

**Figure 4:**
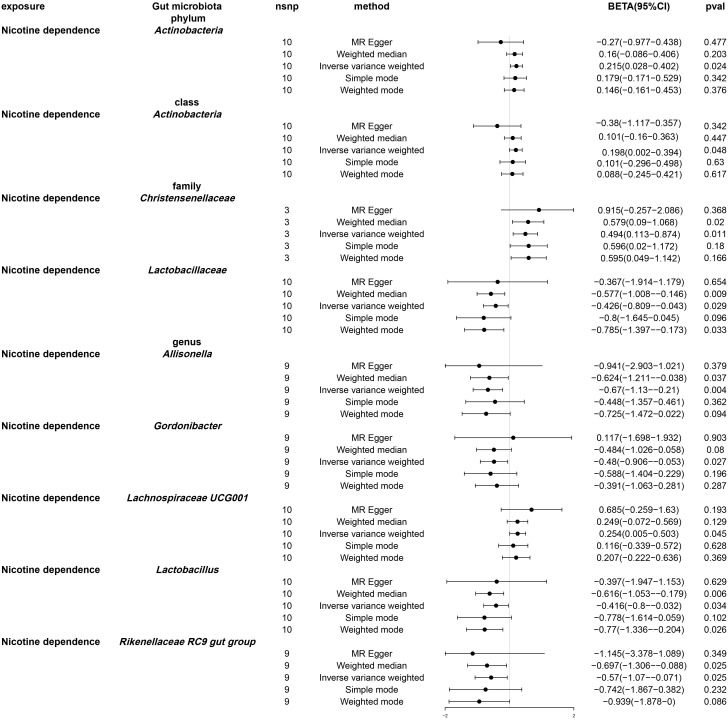
Forest plot of Mendelian randomization of two samples as a result of gut microbiota abundance. Causal effects of five Mendelian randomization methods with nicotine dependence as exposure and gut microbiota abundance as outcome. Effect estimates are expressed as effect size (BETA) with 95% confidence intervals (CI). SNP, single nucleotide polymorphism.

Following univariable Mendelian randomization analysis, a potential causal effect of nicotine dependence on the abundance of five genera, two families, one phylum, and one class was found (**Figure 4**). According to the results of mendelian randomization analysis based on the IVW method, nicotine dependence caused a causal effect on the abundance of *Actinobacteria*, *Christensenellaceae* (beta: 0.494, 95% CI: 0.113-0.874, P = 0.011) and *Lachnospiraceae UCG001* (beta. 0.254, 95% CI: 0.005-0.503, P =0.045) increased in abundance, where the causal effect of nicotine dependence on Actinobacteria was consistent at both the phylum (beta: 0.215, 95% CI: 0.028-0.402, P = 0.024) and class (beta: 0.198, 95% CI: 0.002-0.394, 0.048) levels. Nicotine dependence was simultaneously induced in *Lactobacillaceae* (beta: -0.426, 95% CI: -0.809-0.043, P = 0.029), *Allisonella* (beta: -0.670, 95% CI: -1.130-0.210, P = 0.004), *Gordonibacter* (beta: -0.480, 95% CI: -0.906-0.053, P =0.027), *Lactobacillus* (beta: -0.416, 95% CI: -0.800-0.032, P =0.034), *Rikenellaceae RC9* gut group (beta: -0.570, 95% CI: -1.070-0.071, P =0.025). The causal effects of nicotine dependence on *Lactobacillaceae* were consistent at the family and genus levels. For the phylum *Actinobacteria*, class *Actinobacteria* and genus *Gordonibacter*, MR-Egger method yielded results in the opposite direction to the IVW method, whereas weighted median, simple mode, and weighted mode methods produced analysis results consistent with the IVW method. However, for family *Christensenellaceae*, family *Lactobacillaceae*, genus *Lachnospiraceae UCG001*, genus *Lactobacillus*, genus *Allisonella*, and genus Rikenellaceae RC9, MRegger, weighted median, simple mode, and weighted mode methods provided effect directions consistent with the IVW method. The scatter plot and forest plot were shown in **Figures S1** and **S2**.

**Figure 5:**
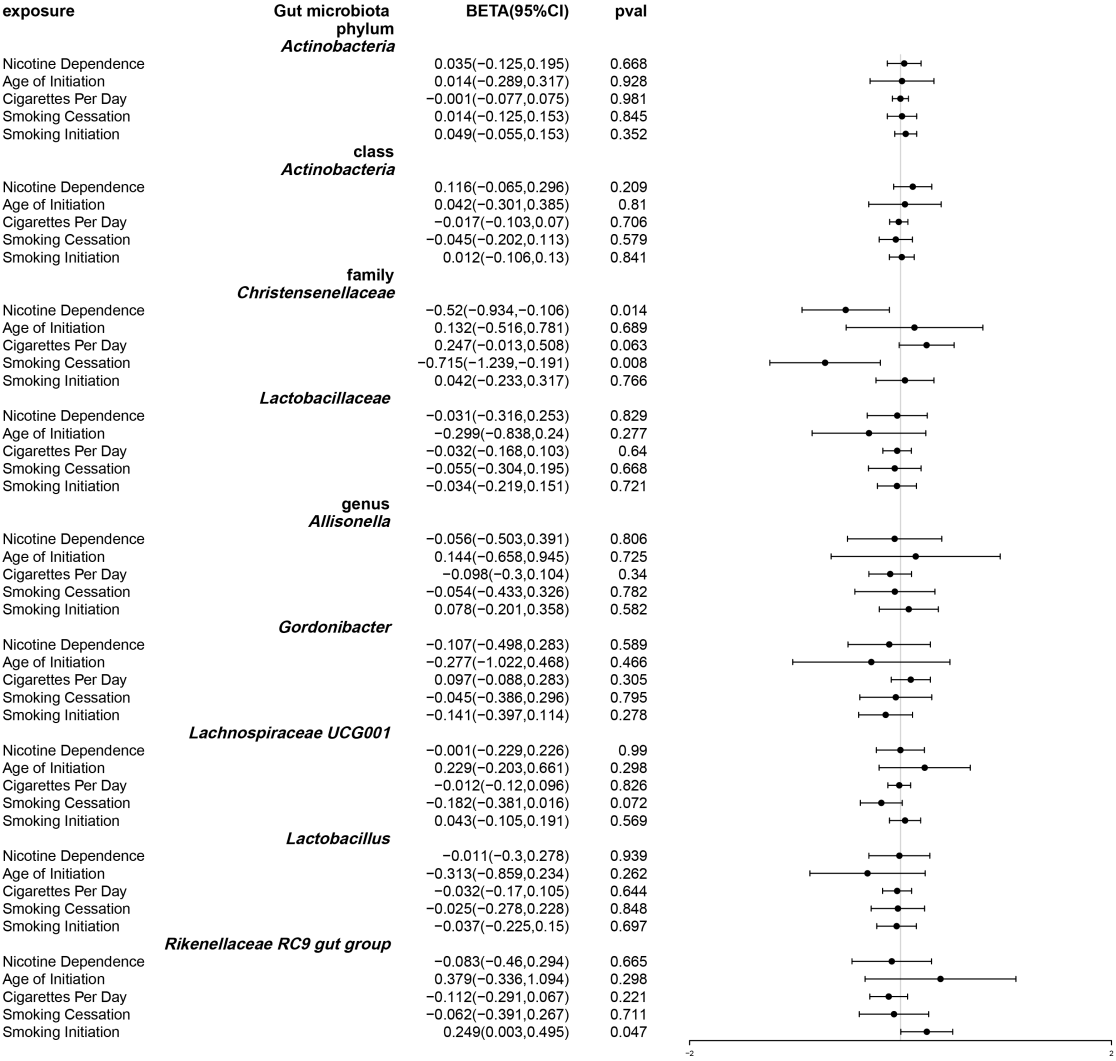
Forest plot of multivariable Mendelian randomization. Causal effects of IVW methods with nicotine dependence and four smoking related phenotypes as exposure while gut microbiota abundance as outcome. Effect estimates are expressed as effect size (BETA) with 95% confidence intervals (CI).

After using multivariable Mendelian randomization to adjust for smoking-related phenotypes (age of smoking initiation, smoking initiation, cigarettes per day, and smoking cessation), nicotine dependence was found to have a causal impact only on *Christensenellaceae* (β: -0.52, 95% CI: -0.934–0.106, P = 0.014). (**Table S5** and **Figure 5**) The detailed information about the instrumental variables used for each covariate in the multivariate Mendelian randomization analysis is recorded in **Table S6**. Furthermore, conditional F statistics of the instrumental variables for each covariate in the multivariable Mendelian randomization were all greater than 10, indicating no weak instrumental bias (**Table S7**).

### Causal effects of gut microbiota on nicotine dependence

In the first step, 1425 SNPs, which were associated with gut microbiota in phylum, class, order, family, and genus, were identified, excluding *Christensenellaceae* since they did not have suitable instrumental variables. The F statistic of each SNP exceeded 10, ranging from 17.17 to 88.41, indicating no weak instrumental bias (**Table S8**). Furthermore, Steiger filtering analysis did not identify any SNPs with opposite causal directions (**Table S9**). We extracted 115 genera, 29 families, 16 orders, and 5 phyla for our instrumental variables. The range of number of IVs from each classification ranged from 3-13.

The significance thresholds for multiple comparisons were set based on the Bonferroni correction: phylum (p = 5.560 × 10^-3^), class (p = 3.125 × 10^-3^), order (p = 2.778 × 10^-3^), family (p = 1.562 × 10^-3^), and genus (p = 4.310 × 10^-4^).

**Figure 6:**
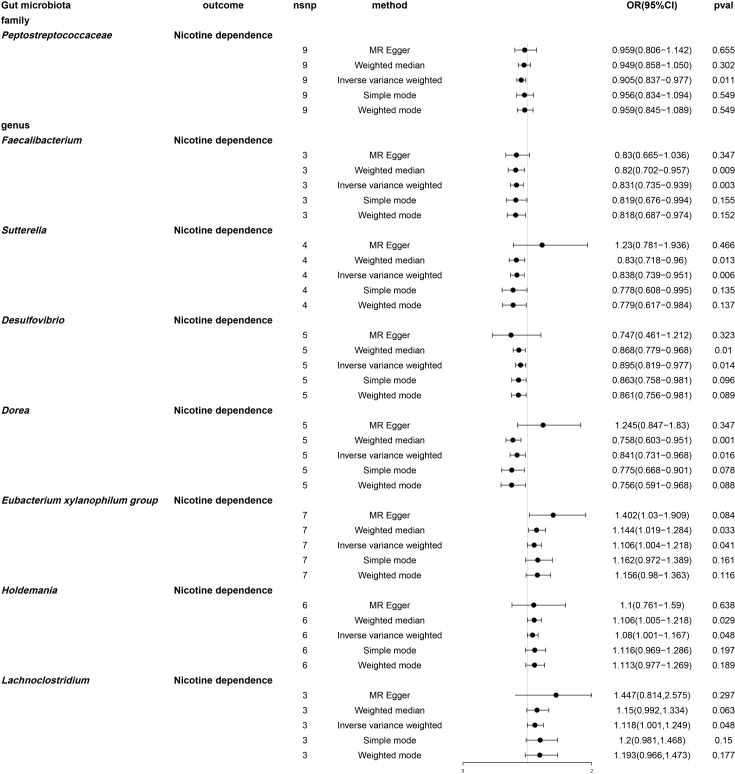
Forest plot of two-sample Mendelian randomization with nicotine dependence as an outcome. Causal effects of five Mendelian randomization methods with gut microbiota abundance as exposure and nicotine dependence as outcome. Effect estimates are expressed as odds ratios (OR) with 95% confidence intervals (CI). SNP, single nucleotide polymorphism.

By using MR analysis (**Table S10**), we combined the SNP effects from the gut microbiota and found that one family and seven genera have a potential causal influence on nicotine dependence (**Figure 6**). According to the IVW approach, the *Eubacterium xylanophilum* group (OR: 1.106, 95% CI: 1.004-1.218, P = 0.041), *Lachnoclostridium* (OR: 1.118, 95% CI: 1.001-1.249, P = 0.048) and *Holdemania* (OR: 1.08, 95% CI: 1.001-1.167, P =0.048) were risk factors for nicotine dependence. Of these, *Lachnoclostridium* had the smallest value of OR. *Peptostreptococcaceae* (OR: 0.905, 95% CI: 0.837-0.977, P =0.011), *Desulfovibrio* (OR: 0.014, 95% CI: 0.819-0.977, P =0.895), *Dorea* (OR: 0.841, 95% CI. 0.731-0.968, P =0.016), *Faecalibacterium* (OR: 0.831, 95% CI: 0.735-0.939, P =0.003) and *Sutterella* (OR: 0.838, 95% CI: 0.739-0.951, P =0.006) were protective factor for nicotine dependence, with *Faecalibacterium* having the smallest value of OR. According to the results of other Mendelian analysis methods, for genus *Sutterella* and genus *Dorea*, MR-Egger method yielded results in the opposite direction to the IVW method, while weighted median, simple mode, and weighted mode methods produced analysis results consistent with the IVW method. For the family *Peptostreptococcaceae*, genus *Eubacterium xylanophilum group*, genus *Lachnoclostridium*, genus *Holdemania*, genus *Lachnoclostridium*, and genus *Desulfovibrio*, MR-Egger, weighted median, simple mode, and weighted mode provided effect directions consistent with the IVW method. The scatter plot and forest plot were shown in **Figures S3** and **S4**.

### Sensitivity analysis

No evidence was found for horizontal pleiotropy when using the MR-Egger regression intercept method on the gut microbiota and nicotine-dependent instrumental variables (**Tables S11**–**S13**). We screened and removed any outliers in the MR-PRESSO analysis and found no horizontal pleiotropy for the gut microbiota or nicotine-dependent instrumental variables (**Tables S14** and **S15**). Furthermore, the majority of Cochrane Q statistics did not show significant heterogeneity (p > 0.05) as shown in the Supplementary Material (**Tables S16**–**S18**). In cases where heterogeneity was found to be significant, we used a random-effects model with an IVW model. The results of leave-one-out sensitivity and funnel plot were shown in **Figures S5**–**S8**.

## Discussion

Our study employed a bidirectional Mendelian randomization approach to assess the causality between nicotine dependence and gut microbial abundance. To our knowledge, this is the first mendelian randomization study to examine the causal relationship between nicotine dependence and gut microbial abundance.

Smoking is a complex behavior that encompasses several stages, including initiation, regular smoking, nicotine dependence, cessation, and relapse. While some individuals may maintain low levels of smoking without experiencing dependence (49), others may become heavily dependent, which escalates the challenges associated with stopping smoking and augments the risk of relapse (50, 51). Furthermore, while smoking behavior broadly encompasses various stages, nicotine dependence has a strong genetic component with high heritability rates (up to 75%) coupled with environmental factors (52). Nicotine dependence is a significant predictor of the severity of tobacco withdrawal (53), response to treatments (54), and smoking-related health outcomes (55, 56), which impede smoking cessation success.

Previous studies have found that smokers are often accompanied by alterations in gut microbiota composition. Proposed mechanisms to explain the impact of smoking on the gut microbiota include enhanced oxidative stress (57), alterations in intestinal tight junctions and gut mucin composition (58), and changes in acid-base homeostasis (59). Smoking leads to changes in the composition of the gut microbiota, showing an increased abundance of certain bacteria such as *Prevotella*, *Veloperae*, *Anaplasma*, *Acidophilus*, and *Helicobacter oxysporum*, and a decrease in the abundance of other bacteria such as *Thiotrichales* and *Helicobacter Lachesis* (60). The use of nicotine products leads to known health consequences, but may also be a major cause of intestinal ecological disorders and increased intestinal permeability (14). Smoking cessation partially reversed these microbial alterations, resulting in increased microbial diversity and abundance in certain phyla. In healthy individuals who quit smoking, significant changes were observed in the fecal microbiota, including an increase in the relative abundance of thick-walled bacteria and actinomycetes and a decrease in anaplasma and amoebae (61). Quitting smoking resulted in significant changes in the fecal microbiota of healthy individuals. Although studies have investigated the effects of tobacco use on gut microbiota, previous studies are limited by small sample sizes and inaccurate assessment of tobacco use. Patient inclusion is often based solely on self-reported smoking history, with unstable criteria for assessing smoking frequency, which renders studies susceptible to confounding factors that may not be excluded.

While a prior mendelian randomization study has investigated the relationship between smoking associated phenotypes and gut microbiota (62), no study has examined the relationship between nicotine dependence and gut microbiota. Given the aforementioned limitations of previous studies on smoking and gut microbiota, further research is needed to address the relationship between nicotine dependence and gut microbiota. Therefore, in this study, we used genome-wide association data from patients with nicotine dependence based on FTND scores to investigate the causal relationship between nicotine dependence and gut microbiota using mendelian randomization.

Studies have shown that smoking leads to a higher abundance of *Streptococcus* spp. and *Desulfovibri* in the digestive tract (63), as well as a higher abundance of *Streptococcus* spp. in the upper small intestinal mucosa of smokers (64). Additionally, smokers show a significantly lower abundance of *Faecalibacterium* in the gut (65). In this study, we found that genetically dependent nicotine dependence can lead to a decrease in the gut abundance of *Christensenellaceae*. Furthermore, even after smoking cessation, the effect of decreased *Christensenellaceae* abundance persists. *Christensenellaceae*, belonging to the *Firmicutes* phylum, is widely present in the human gut and mucosa (66–69), and it plays a crucial role in host health. Previous studies have found a negative correlation between the relative abundance of *Christensenellaceae* and host body mass index in different populations and multiple research studies (70). It is also closely associated with glucose metabolism (71, 72) and inflammatory bowel disease (73). *Christensenellaceae* may serve as probiotics to improve health status (74), but further research is needed to elucidate the underlying mechanisms. Previous studies have shown that smoking reduces the abundance of Firmicutes in the gut (60). Our study suggests a causal effect between genetically predicted nicotine dependence and *Christensenellaceae*. Further investigation is needed to explore the potential of *Christensenellaceae* in improving symptoms of nicotine dependence in patients and preventing diseases associated with nicotine dependence.

From the perspective of the effect of gut microbiota on nicotine dependence, a recent study has shown that colonization of *Bacteroides xylanisolvens*, a human gut bacteria, can effectively degrade intestinal nicotine, providing a new target for the treatment of patients with non-alcoholic fatty liver disease (75). This suggests that gut microbiota abundance may influence nicotine metabolism and further impact the disease progression of nicotine dependence. However, there is insufficient evidence from previous studies to assess whether gut microbiota abundance has a preventive or promotive effect on nicotine dependence. Our study found that *Peptostreptococcaceae*, *Desulfovibrio*, *Dorea*, *Faecalibacterium*, and *Sutterella* decrease the risk of nicotine dependence, while the *Eubacterium xylanophilum* group and *Holdemania* increase the risk of nicotine dependence. These findings have not been widely reported previously, indicating a potential contribution of this study to the existing literature.

There are several advantages in our study. Specifically, we employed a biodirectional mendelian randomization analysis to establish the causal association between gut microbiota and nicotine dependence. This approach allowed us to control for confounding factors and minimize the risk of reverse causation. The gut microbiota and nicotine dependence genome-wide association data were retrieved from the largest available GWAS meta-analysis to ensure the statistical robustness of the instrumental variables used in the Mendelian randomization analysis. To minimize the potential impact of weak IV bias, we employed a suitable threshold for the genomic correlation of instrumental variables (p = 5e-06). This threshold was chosen based on the availability of a sufficient number of SNPs with adequate statistical power for most gut flora, effectively avoiding confounding. In contrast, previous studies used a p-value cut-off of 1e-05 (36, 76) or 1e-06 (62) resulting in only a few gut flora receiving 3 or more SNPs for the Mendelian randomization analysis. Consequently, the power of the previous studies might have been insufficient, introducing false negatives. In addition, the previous study ended up including only 41 gut microbiota (62), which may have circumvented the inclusion of a larger number of flora and led to false positives when performing the FDR test for p-value (FDR = p*n/rank). The phenotypes utilized in the prior Mendelian randomization analyses of smoking initiation, lifetime smoking, and daily cigarette consumption, were not clinically practical for age-specific interventions. In contrast, our study of the FTND scale for diagnosing nicotine dependence as a phenotype may offer clinical guidance to those who smoke, but do not meet the diagnostic criteria for nicotine dependence. Employing the FTND scale may help prevent the development of nicotine dependence.

Although our study has several strengths, we acknowledge some limitations. Notably, the p-values in our findings are not robust to the Bonferroni method adjusted for significance. However, it is important to note that our study is hypothesis-driven, based on strong physiological evidence that supports the epidemiologically established link between gut flora and nicotine dependence. To strengthen the results further, future studies may need to include a larger sample size of nicotine-dependent patients. Additionally, the use of multiple comparisons to adjust p-values may increase the risk of false negatives due to the high number of microbial taxa and the multilevel structure (correlation between abundance and microbial strains) and the correlation between nicotine dependence. Therefore, caution should be exercised when interpreting negative results or potentially significant p-values. As with the previous point, future GWAS studies could benefit from increasing the sample size of patients with gut flora and nicotine dependence to reduce the likelihood of such biases. Third, as the majority of participants in the Nicotine Dependent GWAS were of European ancestry, the external validity of our findings to other ethnic groups may be constrained. Given that smoking is more prevalent among men, a disproportionately high number of male patients were included in the nicotine-dependent phenotype. Moreover, gender differences may exist in the composition of the gut microbiota. In our study, out of the nicotine dependence GWAS data employed, 53.2% of the participants were male, and the relatively even sex ratio could alleviate the potential gender bias to some extent. Nevertheless, the summary data from genome-wide association analyses limited our capacity to conduct further subgroup analyses to explore gender-specific discrepancies.

## Conclusion

In conclusion, our investigation confirms the causal link between genetically predicted nicotine dependence and gut microbiota, underscoring the interactive impacts of nicotine dependence on gut microbes that might act as novel biomarkers and yield revelations for addressing and avoiding nicotine dependence.

## Data availability statement

Publicly available datasets were analyzed in this study. This data can be found here: The GWAS meta-analysis results on nicotine dependence were provided by the dbGaP study of PubMed (study_id=phs001532) and can be downloaded from http://www.ncbi.nlm.nih.gov/projects/gap/cgi-bin/study.cgi?study_id=phs001532.v1.p1. The summary data on gut microbiota is from MiBioGen consortium, which can be obtained from the IEU GWAS database (https://gwas.mrcieu.ac.uk/) (GWAS ID: ebi-a-GCST90016908– ebi-a-GCST90017118).The summary data on smoking related phenotypes is from GSCAN consortium, which can be obtained from the University Digital Conservancy Home (https://conservancy.umn.edu/handle/11299/201564).

## Ethics statement

Ethical approval was not required for the study involving humans in accordance with the local legislation and institutional requirements. Written informed consent to participate in this study was not required from the participants or the participants’ legal guardians/next of kin in accordance with the national legislation and the institutional requirements.

## Author contributions

All authors have been involved in the preparation of the manuscript and approved its submission. Conceptualization: LL, ZC and JZ. Formal analysis: YC, MZ, KJ and JL. Funding acquisition: LL, ZC and JZ. Methodology: JL, SW and LL. Writing original draft: YC, MZ and KJ. Writing-review and editing: YC, MZ, KJ, JL, SW, LL, ZC and JZ. All authors contributed to the article and approved the submitted version.
