# Supplementary material for: Association of nicotine dependence and gut microbiota: a bidirectional two-sample Mendelian randomization study
Source: Front Immunol. 2023 Nov 7;14:1244272. doi: 10.3389/fimmu.2023.1244272 (PMC10664251; doi:10.3389/fimmu.2023.1244272)
Supplement: Supplementary file 1 [file Table_1.docx]

**STROBE-MR checklist of recommended items to address in reports of Mendelian randomization studies**^1^ ^2^

| **Item No.** | **Section** | **Checklist item** | **Page No.** | **Relevant text from manuscript** |
| --- | --- | --- | --- | --- |
| 1 | **TITLE and ABSTRACT** | Indicate Mendelian randomization (MR) as the study’s design in the title and/or the abstract if that is a main purpose of the study | 1,2 | Title: Association of Nicotine Dependence and Gut Microbiota: A Bidirectional Two-sample Mendelian Randomization Study  Abstract:  Methods: A two-sample bidirectional Mendelian randomization (MR) study was conducted using the largest existing gut microbiota and nicotine dependence genome-wide association studies (GWAS). |
|  | **INTRODUCTION** |  |  |  |
| 2 | **Background** | Explain the scientific background and rationale for the reported study. What is the exposure? Is a potential causal relationship between exposure and outcome plausible? Justify why MR is a helpful method to address the study question | 3,4 | Introduction: Smoking can modify the microbiome in several regions(Savin et al., 2018), including the periodontal, intestinal, and respiratory tracts, and augments the mechanisms whereby changes in mucosal immune responses, fluctuations in intestinal cytokine levels, alteration in intestinal permeability, and epigenetic modification alter gene expression(Kaur et al., 2019; Papoutsopoulou et al., 2020). Prebiotics are undigestible food elements that can selectively promote the growth and function of the colonic microbiota, ultimately improving host health(Patra et al., 2022). Supplementation of probiotics and the reconstruction of a healthy microbiota in the gut are now considered effective strategies for treating diseases caused by gut microbiota dysbiosis (Hemarajata and Versalovic, 2013; Erejuwa et al., 2014; Das et al., 2022). Therefore, using appropriate prebiotics to target specific microbial communities may be an effective approach for preventing and treating nicotine dependence. However, the causal relationship and mechanisms between gut microbiota and nicotine dependence are still unclear, which poses obstacles to the prevention and treatment of nicotine dependence. Thus, it is imperative to study the causal link between the gut microbiota and nicotine dependence. |
| 3 | **Objectives** | State specific objectives clearly, including pre-specified causal hypotheses (if any). State that MR is a method that, under specific assumptions, intends to estimate causal effects | 4 | Introduction: Building upon the recent large-scale genome-wide association studies (GWAS) on the gut microbiota (Bonder et al., 2016; Goodrich et al., 2016; Wang et al., 2016) and disease, we employed the Mendelian randomization approach to investigate the causal link between the gut microbiota and the risk of nicotine dependence in this study. This study aims to explore the impact of genetic prediction of nicotine dependence on the gut microbiota, and elucidate the role of the gut microbiota in the pathogenesis of nicotine dependence through genetic prediction. |
|  | **METHODS** |  |  |  |
| 4 | **Study design and data sources** | Present key elements of the study design early in the article. Consider including a table listing sources of data for all phases of the study. For each data source contributing to the analysis, describe the following: |  | table listing sources of data for all phases of the study: Table S2 |
|  | a) | Setting: Describe the study design and the underlying population, if possible. Describe the setting, locations, and relevant dates, including periods of recruitment, exposure, follow-up, and data collection, when available. | 4,5 | GWAS SUMMARY DATA FOR NICOTINE DEPENDENCE  The authors conducted a genome-wide meta-analysis on 38,602 former smokers of European and African American descent with mild (N = 17,796; 46.1%), moderate (N = 13,527; 35%), or severe (N = 7,279; 18.9%) nicotine dependence.  GWAS SUMMARY DATA FOR GUT MICROBIOTA  Data on the composition of human gut microbiota were obtained from the MiBioGen consortium through a large-scale multi-ethnic GWAS study(Kurilshikov et al., 2021). This study involved 18,340 participants from 24 cohorts in countries such as the United States, Canada, Israel, Korea, Germany, Denmark, the Netherlands, Belgium, Sweden, Finland, and the UK.  GWAS SUMMARY DATA FOR SMOKING-RELATED PHENOTYPES  The GWAS data for smoking-related phenotypes were obtained from a meta-analysis of GWAS summary association data from 1,232,091 individuals predominantly of European ancestry. |
|  | b) | Participants: Give the eligibility criteria, and the sources and methods of selection of participants. Report the sample size, and whether any power or sample size calculations were carried out prior to the main analysis | 4,5 | GWAS SUMMARY DATA FOR NICOTINE DEPENDENCE  Genotyping was performed on various genome-wide platforms, and after quality control, 1000G genomic interpolation was used to analyze the genotype data. Linear regression was carried out on the data and adjusted for age, sex, pedigree principal components, and cohort-specific covariates. The Genome-wide association study (GWAS) results were combined using METAL with fixed-effects inverse variance weighting meta-analysis, across all studies with FTND data to maximize statistical power. More than 99% of the former smokers were over 18 years old, and in case of the presence of relatives, family structure was adjusted.  GWAS SUMMARY DATA FOR GUT MICROBIOTA  The participants' 16S ribosomal RNA gene sequences and genotyped data were analyzed to investigate the relationship between human autosomal genetic variation and gut microbial communities.  GWAS SUMMARY DATA FOR SMOKING-RELATED PHENOTYPES  The authors applied extensive genetic quality control and filtering to the summary statistics provided by each cohort. Imputed variants with an imputation quality below 0.3 (estimated squared correlation between imputed and true dosage) were subsequently removed. Then, the allele labels and allele frequencies of each study were compared with those of the imputation reference panel, and discrepancies were either removed or harmonized. Finally, a meta-analysis was conducted using the software package rareGWAMA based on a fixed-effect model. |
|  | c) | Describe measurement, quality control and selection of genetic variants | 4,5 | GWAS SUMMARY DATA FOR NICOTINE DEPENDENCE  Genotyping was performed on various genome-wide platforms, and after quality control, 1000G genomic interpolation was used to analyze the genotype data. Linear regression was carried out on the data and adjusted for age, sex, pedigree principal components, and cohort-specific covariates. The Genome-wide association study (GWAS) results were combined using METAL with fixed-effects inverse variance weighting meta-analysis, across all studies with FTND data to maximize statistical power.  GWAS SUMMARY DATA FOR GUT MICROBIOTA  The participants' 16S ribosomal RNA gene sequences and genotyped data were analyzed to investigate the relationship between human autosomal genetic variation and gut microbial communities. The study included 211 taxa comprising 35 families, 20 orders, 16 phyla, 9 orders, and 131 genera.  GWAS SUMMARY DATA FOR SMOKING-RELATED PHENOTYPES  The authors applied extensive genetic quality control and filtering to the summary statistics provided by each cohort. Imputed variants with an imputation quality below 0.3 (estimated squared correlation between imputed and true dosage) were subsequently removed. Then, the allele labels and allele frequencies of each study were compared with those of the imputation reference panel, and discrepancies were either removed or harmonized. Finally, a meta-analysis was conducted using the software package rareGWAMA based on a fixed-effect model. |
|  | d) | For each exposure, outcome, and other relevant variables, describe methods of assessment and diagnostic criteria for diseases | 4 | The authors conducted a genome-wide meta-analysis on 38,602 former smokers of European and African American descent with mild (N = 17,796; 46.1%), moderate (N = 13,527; 35%), or severe (N = 7,279; 18.9%) nicotine dependence. |
|  | e) | Provide details of ethics committee approval and participant informed consent, if relevant | 7 | Standard Protocol Approval, Registration and Patient Consent  The GWAS data used in this study were all from publicly available databases. The summary statistics of nicotine dependence, smoking-related phenotypes and gut microbiota abundance do not contain any personal information, and each GWAS has received ethical approval from the relevant ethical review board. |
| 5 | **Assumptions** | Explicitly state the three core IV assumptions for the main analysis (relevance, independence and exclusion restriction) as well assumptions for any additional or sensitivity analysis | 4 | STUDY DESIGN  The aim of this study was to evaluate the causal relationship between genetically predicted nicotine dependence risk and genetically predicted abundance of gut microbiota using a Mendelian randomization method. The Mendelian randomization design consisted of three components. Firstly, the selection of genetic variants as instrumental variables for nicotine dependence. Secondly, the acquisition of a summary dataset for genetic instruments derived from a genome-wide association study of nicotine dependence, and finally, obtaining a summary dataset for single nucleotide polymorphism results. These results were used to investigate the impact of GWAS genetic instruments on gut microbiota. Fig. 1 outlines the design of the Mendelian randomization study, while Fig. 2 presents an overview of the investigation along with a flow chart. |
| 6 | **Statistical methods: main analysis** | Describe statistical methods and statistics used |  |  |
|  | a) | Describe how quantitative variables were handled in the analyses (i.e., scale, units, model) | 6 | The initial analysis utilized inverse variance weighting (IVW) to explore the possible causal relationship between gut microbiota abundance and nicotine dependence. |
|  | b) | Describe how genetic variants were handled in the analyses and, if applicable, how their weights were selected | 5,6 | SELECTION OF INSTRUMENTAL VARIABLES  This study aimed to explore the causal relationship between nicotine dependence and gut microbiota through the Mendelian Randomization analysis of instrumental variables. First, single nucleotide polymorphisms (SNPs) with a genome-wide significance threshold (5 × 10-8) were selected as instrumental variables (IVs) relating to nicotine dependence. After linkage disequilibrium analysis (R2 < 0.001, clumping distance = 10,000 kb), only one SNP was retained. To ensure a satisfactory number of IVs, a significance threshold of 5 × 10-6 for SNP versus nicotine-dependent phenotypes and a minor allele frequency (MAF) threshold of 0.01 were set. Additional linkage disequilibrium analysis (R2 < 0.001, clumping distance = 10,000 kb) was conducted on the European 1000 Genomes Project data to screen out instrumental variables that could cause biased results.  Steiger filtering analysis(Hemani et al., 2017) was further used to determine the directional effect of individual instrumental variable SNPs on the outcome. A "TRUE" result predicts the expected direction of association. SNPs that are shown as "False" in the Steiger filtering analysis will be excluded and not included in the subsequent Mendelian randomization analysis. |
|  | c) | Describe the MR estimator (e.g. two-stage least squares, Wald ratio) and related statistics. Detail the included covariates and, in case of two-sample MR, whether the same covariate set was used for adjustment in the two samples | 6 | Two-Sample Mendelian randomization  The initial analysis utilized inverse variance weighting (IVW) to explore the possible causal relationship between gut microbiota abundance and nicotine dependence.  Additionally, for mendelian randomization analysis, the weighted median, MR-Egger, simple mode, and weighted mode methods were used. |
|  | d) | Explain how missing data were addressed | 4,5 | Genotyping was performed on various genome-wide platforms, and after quality control, 1000G genomic interpolation was used to analyze the genotype data. Linear regression was carried out on the data and adjusted for age, sex, pedigree principal components, and cohort-specific covariates.  Imputed variants with an imputation quality below 0.3 (estimated squared correlation between imputed and true dosage) were subsequently removed. Then, the allele labels and allele frequencies of each study were compared with those of the imputation reference panel, and discrepancies were either removed or harmonized. Finally, a meta-analysis was conducted using the software package rareGWAMA based on a fixed-effect model. |
|  | e) | If applicable, indicate how multiple testing was addressed | 6 | A multiple test significance threshold was set at 0.05/n (where n represents the number of independent bacterial taxa at the corresponding taxonomic level) due to the numerous comparisons that took place at each character level, such as phylum, class, order, family, and genus. Significance values that fell between the multiple test significance threshold and 0.05 were considered potentially significant. |
| 7 | **Assessment of assumptions** | Describe any methods or prior knowledge used to assess the assumptions or justify their validity | 6 | We evaluated the strength of instrumental variables by computing the F-statistic as F = R2 × (N - 2)/(1 - R2), where R2 represents the proportion of variation in the exposure factor clarified by each instrumental variable while N represents the sample size for the GWAS that relates to the exposure(Burgess et al., 2011). In turn, R2 is calculated as (2 x EAF x (1 - EAF) x beta2)/[(2 x EAF x (1 - EAF) x beta2) + (2 x EAF x (1 - EAF) x N x SE(beta)2)], where EAF is the effect allele frequency, beta is the estimated genetic effect of the exposure factor, Ν is the GWAS sample size for the SNP-exposure correlation, and SE (beta) refers to the genetic effect’s standard error(Shim et al., 2015). Instrumental variables having a F-statistic <10 for weak instruments may suggest a possible bias and need to be removed. Meanwhile, those having a F-statistic >10 are included for further analysis. |
| 8 | **Sensitivity analyses and additional analyses** | Describe any sensitivity analyses or additional analyses performed (e.g. comparison of effect estimates from different approaches, independent replication, bias analytic techniques, validation of instruments, simulations) | 7 | We conducted several sensitivity analyses consisting of tests such as Cochran's Q statistic, funnel plots, leave-one-out analysis, and the MR-Egger intercept test. Cochran's Q test revealed heterogeneity in the instrumental variables in case the p-value was lower than 0.05. The "leave-one-out" method was applied to validate the causal relationship between nicotine dependency and gut microbiota abundance.  For detecting and correcting pleiotropic outliers, we employed the mendelian randomized pleiotropic residuals and outliers (MR-PRESSO) method(Verbanck et al., 2018). |
| 9 | **Software and pre-registration** |  |  |  |
|  | a) | Name statistical software and package(s), including version and settings used | 6 | Statistical analyses were performed using R software version 4.2.2, utilizing the R packages "TwoSampleMR" (v.0.5.6)(Hemani et al., 2017), "MRPRESSO" (v.1.0)(Verbanck M, 2017), and "MendelianRandomization"(OYJS, 2023) (v.0.7.0) in order to carry out a Mendelian randomization (MR) analysis on the causal relationship between nicotine dependence and gut microbiota. |
|  | b) | State whether the study protocol and details were pre-registered (as well as when and where) |  | This study protocol and details were not pre-registered. |
|  | **RESULTS** |  |  |  |
| 10 | **Descriptive data** |  |  |  |
|  | a) | Report the numbers of individuals at each stage of included studies and reasons for exclusion. Consider use of a flow diagram |  | The GWAS data used in this study is derived from the GWAS meta-analysis data of existing studies, so it is not reported. |
|  | b) | Report summary statistics for phenotypic exposure(s), outcome(s), and other relevant variables (e.g. means, SDs, proportions) |  | We report summary statistics for nicotine dependence, smoking-related phenotypes, and gut microbiota data in Supplementary Table S1. |
|  | c) | If the data sources include meta-analyses of previous studies, provide the assessments of heterogeneity across these studies |  | We present the I-squared values of the GWAS meta-results for instrumental variables for nicotine dependence, smoking-related phenotype data in Supplementary Table S2 and Supplementary Table 17. |
|  | d) | For two-sample MR:  i.  Provide justification of the similarity of the genetic variant-exposure associations between the exposure and outcome samples  ii.  Provide information on the number of individuals who overlap between the exposure and outcome studies | 7,8 | 17 SNPs met the instrumental variable screening criteria for nicotine dependence, and all had an F-statistic >10 indicating no weak instrumental bias (Table S2). In addition, Steiger filtering analysis helped to exclude SNPs with reverse causal directions (three from genus Victivallis and one from genus Prevotella9) (Table S3).  In the first step, 1425 SNPs, which were associated with gut microbiota in phylum, class, order, family, and genus, were identified, excluding Christensenellaceae since they did not have suitable instrumental variables. The F statistic of each SNP exceeded 10, indicating no weak instrumental bias (Table S6). Furthermore, Steiger filtering analysis did not identify any SNPs with opposite causal directions (Table S7). We extracted 115 genera, 29 families, 16 orders, and 5 phyla for our instrumental variables. The range of IVs from each classification ranged from 3-13. |
| 11 | **Main results** |  |  |  |
|  | a) | Report the associations between genetic variant and exposure, and between genetic variant and outcome, preferably on an interpretable scale |  | Table S2, Table S6 |
|  | b) | Report MR estimates of the relationship between exposure and outcome, and the measures of uncertainty from the MR analysis, on an interpretable scale, such as odds ratio or relative risk per SD difference |  | Table S4, Table S5, Table S8 |
|  | c) | If relevant, consider translating estimates of relative risk into absolute risk for a meaningful time period |  | Not applicable. |
|  | d) | Consider plots to visualize results (e.g. forest plot, scatterplot of associations between genetic variants and outcome versus between genetic variants and exposure) |  | Figure S1 and Figure S5 are scatterplot of associations between genetic variants and outcome versus between genetic variants and exposure;  Figure S2 and Figure S6 are forest plot of associations between genetic variants and outcome versus between genetic variants and exposure. |
| 12 | **Assessment of assumptions** |  |  |  |
|  | a) | Report the assessment of the validity of the assumptions | 7 | 17 SNPs met the instrumental variable screening criteria for nicotine dependence, and all had an F-statistic >10 indicating no weak instrumental bias (Table S2). |
|  | b) | Report any additional statistics (e.g., assessments of heterogeneity across genetic variants, such as *I^2^*, Q statistic or E-value) | 9 | No evidence was found for horizontal pleiotropy when using the MR-Egger regression intercept method on the gut microbiota and nicotine-dependent instrumental variables (Table S12, Table S13 and Table S14). We screened and removed any outliers in the MR-PRESSO analysis and found no horizontal pleiotropy for the gut microbiota or nicotine-dependent instrumental variables (Table S15 and Table S16). Furthermore, the majority of Cochrane Q statistics did not show significant heterogeneity (p > 0.05) as shown in the Supplementary Material (Table S9, Table S10 and Table S11). In cases where heterogeneity was found to be significant, we used a random-effects model with an IVW model. |
| 13 | **Sensitivity analyses and additional analyses** |  |  |  |
|  | a) | Report any sensitivity analyses to assess the robustness of the main results to violations of the assumptions |  | In order to enhance the robustness of the analysis, we have supplemented Mendelian randomization (MR) results using four methods: MR Egger, Simple mode, Weighted median, Weighted mode, as shown in Supplementary Tables S4 and S8. |
|  | b) | Report results from other sensitivity analyses or additional analyses |  | We have provided the results of Mendelian randomization using the MR-PRESSO method in Supplementary Tables S15 and S16. |
|  | c) | Report any assessment of direction of causal relationship (e.g., bidirectional MR) | 7 | Reverse Mendelian randomization analysis  To investigate whether gut microbiota abundance is associated with the risk of nicotine dependence, we conducted a reverse Mendelian randomization (MR) analysis using SNPs related to gut microbiota abundance as instrumental variables (where gut microbiota is the exposure and nicotine dependence risk is the outcome). |
|  | d) | When relevant, report and compare with estimates from non-MR analyses | 10 | In this study, we found that genetically dependent nicotine dependence can lead to a decrease in the gut abundance of Christensenellaceae.  Previous studies have shown that smoking reduces the abundance of Firmicutes in the gut(Shapiro et al., 2022). |
|  | e) | Consider additional plots to visualize results (e.g., leave-one-out analyses) |  | We have provided the results of the Leave-one-out analysis in Supplementary Figures S3 and S7. Additionally, the analysis results of funnel plots are presented in Supplementary Figures S4 and S8. |
|  | **DISCUSSION** |  |  |  |
| 14 | **Key results** | Summarize key results with reference to study objectives | 9 | Our study employed a bidirectional Mendelian randomization approach to assess the causality between nicotine dependence and gut microbial abundance. To our knowledge, this is the first mendelian randomization study to examine the causal relationship between nicotine dependence and gut microbial abundance. |
| 15 | **Limitations** | Discuss limitations of the study, taking into account the validity of the IV assumptions, other sources of potential bias, and imprecision. Discuss both direction and magnitude of any potential bias and any efforts to address them | 11,12 | Although our study has several strengths, we acknowledge some limitations. Notably, the p-values in our findings are not robust to the Bonferroni method adjusted for significance. However, it is important to note that our study is hypothesis-driven, based on strong physiological evidence that supports the epidemiologically established link between gut flora and nicotine dependence. To strengthen the results further, future studies may need to include a larger sample size of nicotine-dependent patients. Additionally, the use of multiple comparisons to adjust p-values may increase the risk of false negatives due to the high number of microbial taxa and the multilevel structure (correlation between abundance and microbial strains) and the correlation between nicotine dependence. Therefore, caution should be exercised when interpreting negative results or potentially significant p-values. As with the previous point, future GWAS studies could benefit from increasing the sample size of patients with gut flora and nicotine dependence to reduce the likelihood of such biases. Third, as the majority of participants in the Nicotine Dependent GWAS were of European ancestry, the external validity of our findings to other ethnic groups may be constrained. Given that smoking is more prevalent among men, a disproportionately high number of male patients were included in the nicotine-dependent phenotype. Moreover, gender differences may exist in the composition of the gut microbiota. In our study, out of the nicotine dependence GWAS data employed, 53.2% of the participants were male, and the relatively even sex ratio could alleviate the potential gender bias to some extent. Nevertheless, the summary data from genome-wide association analyses limited our capacity to conduct further subgroup analyses to explore gender-specific discrepancies. |
| 16 | **Interpretation** |  |  |  |
|  | a) | Meaning: Give a cautious overall interpretation of results in the context of their limitations and in comparison with other studies | 11 | Specifically, we employed a biodirectional mendelian randomization analysis to establish the causal association between gut microbiota and nicotine dependence. This approach allowed us to control for confounding factors and minimize the risk of reverse causation. The gut microbiota and nicotine dependence genome-wide association data were retrieved from the largest available GWAS meta-analysis to ensure the statistical robustness of the instrumental variables used in the Mendelian randomization analysis. To minimize the potential impact of weak IV bias, we employed a suitable threshold for the genomic correlation of instrumental variables (p = 5e-06). This threshold was chosen based on the availability of a sufficient number of SNPs with adequate statistical power for most gut flora, effectively avoiding confounding. In contrast, previous studies used a p-value cut-off of 1e-05(Kurilshikov et al., 2021; Liu et al., 2022) or 1e-06(Fan et al., 2023) resulting in only a few gut flora receiving 3 or more SNPs for the Mendelian randomization analysis. Consequently, the power of the previous studies might have been insufficient, introducing false negatives. In addition, the previous study ended up including only 41 gut microbiota(Fan et al., 2023), which may have circumvented the inclusion of a larger number of flora and led to false positives when performing the FDR test for p-value (FDR = p*n/rank). The phenotypes utilized in the prior Mendelian randomization analyses of smoking initiation, lifetime smoking, and daily cigarette consumption, were not clinically practical for age-specific interventions. In contrast, our study of the FTND scale for diagnosing nicotine dependence as a phenotype may offer clinical guidance to those who smoke, but do not meet the diagnostic criteria for nicotine dependence. Employing the FTND scale may help prevent the development of nicotine dependence. |
|  | b) | Mechanism: Discuss underlying biological mechanisms that could drive a potential causal relationship between the investigated exposure and the outcome, and whether the gene-environment equivalence assumption is reasonable. Use causal language carefully, clarifying that IV estimates may provide causal effects only under certain assumptions | 9,10 | Previous studies have found that smokers are often accompanied by alterations in gut microbiota composition. Proposed mechanisms to explain the impact of smoking on the gut microbiota include enhanced oxidative stress(Wang et al., 2012), alterations in intestinal tight junctions and gut mucin composition(Allais et al., 2016), and changes in acid-base homeostasis(Tomoda et al., 2011).  In this study, we found that genetically dependent nicotine dependence can lead to a decrease in the gut abundance of Christensenellaceae. Furthermore, even after smoking cessation, the effect of decreased Christensenellaceae abundance persists. Christensenellaceae, belonging to the Firmicutes phylum, is widely present in the human gut and mucosa(Huang et al., 2010; Moreno-Indias et al., 2016; Burns et al., 2018; Zakrzewski et al., 2019), and it plays a crucial role in host health. Previous studies have found a negative correlation between the relative abundance of Christensenellaceae and host body mass index in different populations and multiple research studies(Goodrich et al., 2014). It is also closely associated with glucose metabolism(Lim et al., 2017; Lippert et al., 2017) and inflammatory bowel disease(Mancabelli et al., 2017). |
|  | c) | Clinical relevance: Discuss whether the results have clinical or public policy relevance, and to what extent they inform effect sizes of possible interventions | 10 | Christensenellaceae may serve as probiotics to improve health status (Chang et al., 2019), but further research is needed to elucidate the underlying mechanisms. Previous studies have shown that smoking reduces the abundance of Firmicutes in the gut(Shapiro et al., 2022). Our study suggests a causal effect between genetically predicted nicotine dependence and Christensenellaceae. Further investigation is needed to explore the potential of Christensenellaceae in improving symptoms of nicotine dependence in patients and preventing diseases associated with nicotine dependence. |
| 17 | **Generalizability** | Discuss the generalizability of the study results (a) to other populations, (b) across other exposure periods/timings, and (c) across other levels of exposure | 12 | Third, as the majority of participants in the Nicotine Dependent GWAS were of European ancestry, the external validity of our findings to other ethnic groups may be constrained. Moreover, gender differences may exist in the composition of the gut microbiota. In our study, out of the nicotine dependence GWAS data employed, 53.2% of the participants were male, and the relatively even sex ratio could alleviate the potential gender bias to some extent. Nevertheless, the summary data from genome-wide association analyses limited our capacity to conduct further subgroup analyses to explore gender-specific discrepancies. |
|  | **OTHER INFORMATION** |  |  |  |
| 18 | **Funding** | Describe sources of funding and the role of funders in the present study and, if applicable, sources of funding for the databases and original study or studies on which the present study is based |  | Funding information  This research was supported by Guangzhou Municipal Science and Technology Bureau (Grant No. 202102010505 and 202201011206),  the Traditional Chinese Medicine Bureau of Guangdong Province (Grant No. 20211128). These funding organizations did not  participate in the study design, data collection, analysis, interpretation or writing of the manuscript or in the decision to submit  the manuscript for publication. |
| 19 | **Data and data sharing** | Provide the data used to perform all analyses or report where and how the data can be accessed, and reference these sources in the article. Provide the statistical code needed to reproduce the results in the article, or report whether the code is publicly accessible and if so, where |  | Generated Statement: Publicly available datasets were analyzed in this study. This data can be found here: The GWAS meta-analysis results on nicotine dependence were provided by the dbGaP study of PubMed (study_id=phs001532) and can be downloaded from http://www.ncbi.nlm.nih.gov/projects/gap/cgi-bin/study.cgi?study_id=phs001532.v1.p1. The summary data on gut microbiota is from MiBioGen consortium, which can be obtained from the IEU GWAS database (https://gwas.mrcieu.ac.uk/) (GWAS ID: ebi-a-GCST90016908-- ebi-a-GCST90017118). The summary data on smoking related phenotypes is from GSCAN consortium, which can be obtained from the University Digital Conservancy Home (https://conservancy.umn.edu/handle/11299/201564). |
| 20 | **Conflicts of Interest** | All authors should declare all potential conflicts of interest |  | Conflict of interest statement  The authors declare that the research was conducted in the absence of any commercial or financial  relationships that could be construed as a potential conflict of interest |

This checklist is copyrighted by the Equator Network under the Creative Commons Attribution 3.0 Unported (CC BY 3.0) license.

1. Skrivankova VW, Richmond RC, Woolf BAR, Yarmolinsky J, Davies NM, Swanson SA, et al. Strengthening the Reporting of Observational Studies in Epidemiology using Mendelian Randomization (STROBE-MR) Statement. JAMA. 2021;under review.

2. Skrivankova VW, Richmond RC, Woolf BAR, Davies NM, Swanson SA, VanderWeele TJ, et al. Strengthening the Reporting of Observational Studies in Epidemiology using Mendelian Randomisation (STROBE-MR): Explanation and Elaboration. BMJ. 2021;375:n2233.
