# Supplementary material for: Association of nicotine dependence and gut microbiota: a bidirectional two-sample Mendelian randomization study
Source: Front Immunol. 2023 Nov 7;14:1244272. doi: 10.3389/fimmu.2023.1244272 (PMC10664251; doi:10.3389/fimmu.2023.1244272)

**Figure S1** Scatterplot of results of Mendelian randomization with nicotine dependence as exposure and gut microbiota abundance as outcome. (Only results with significant p-values in the IVW method are shown)


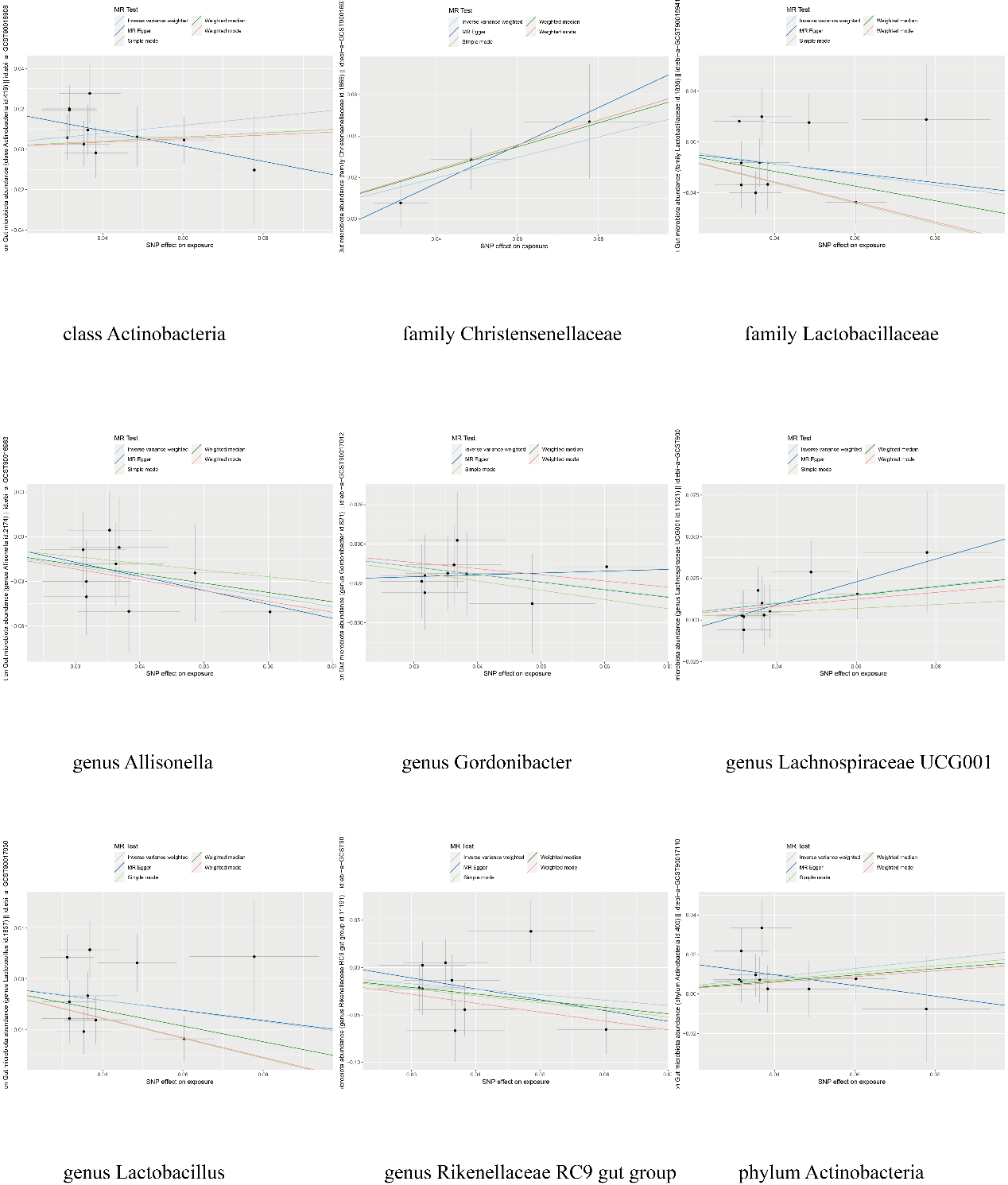


**Figure S2** Forest plot of single SNP analysis using Wald ratios, with nicotine dependence as exposure and gut microbiota abundance as outcome. (Only results with significant p-values in the IVW method are shown)


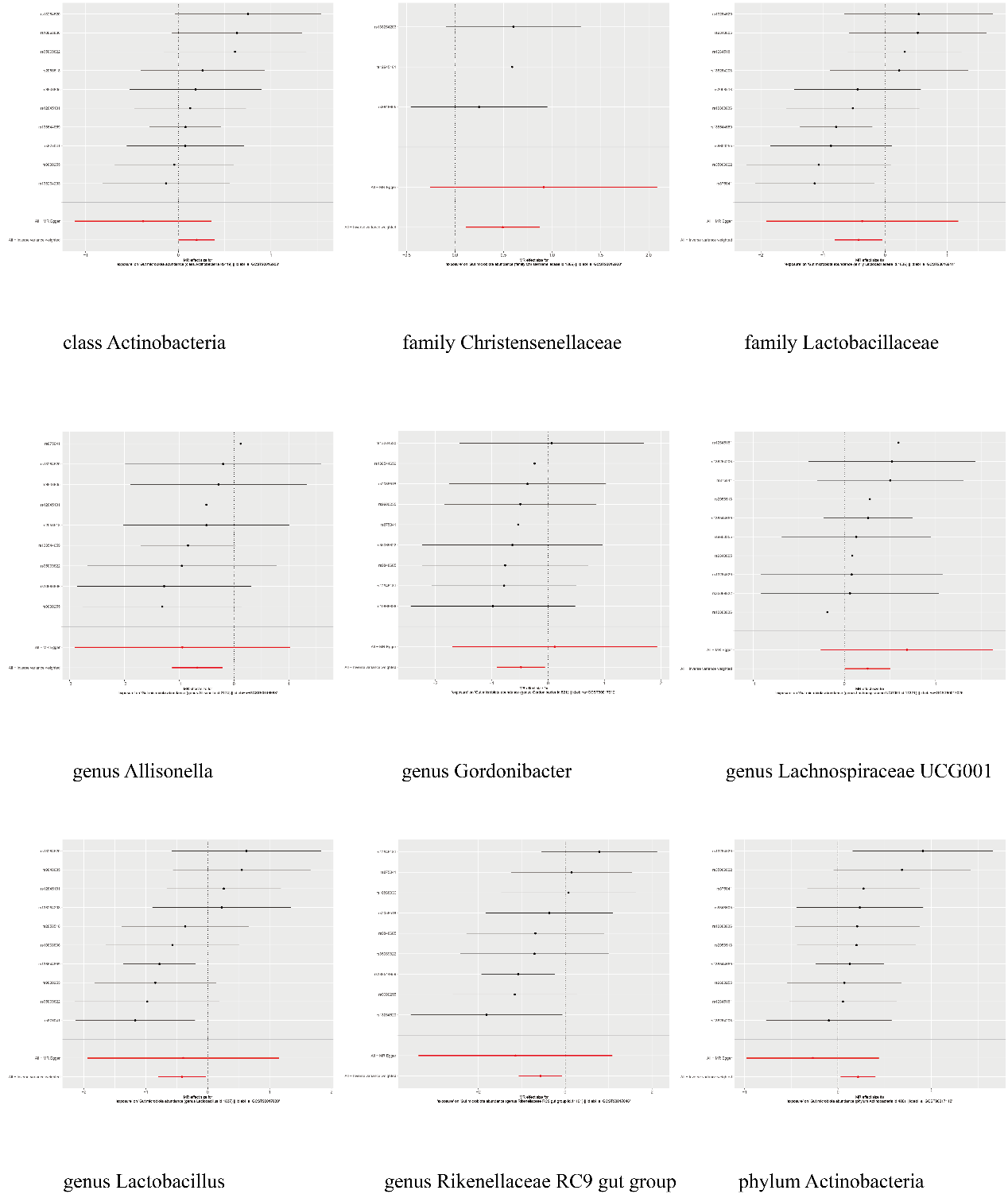


**Figure S3** Leave-one-out analysis plot, with nicotine dependence as exposure and gut microbiota abundance as result. (Only results with significant p-values in the IVW method are shown)


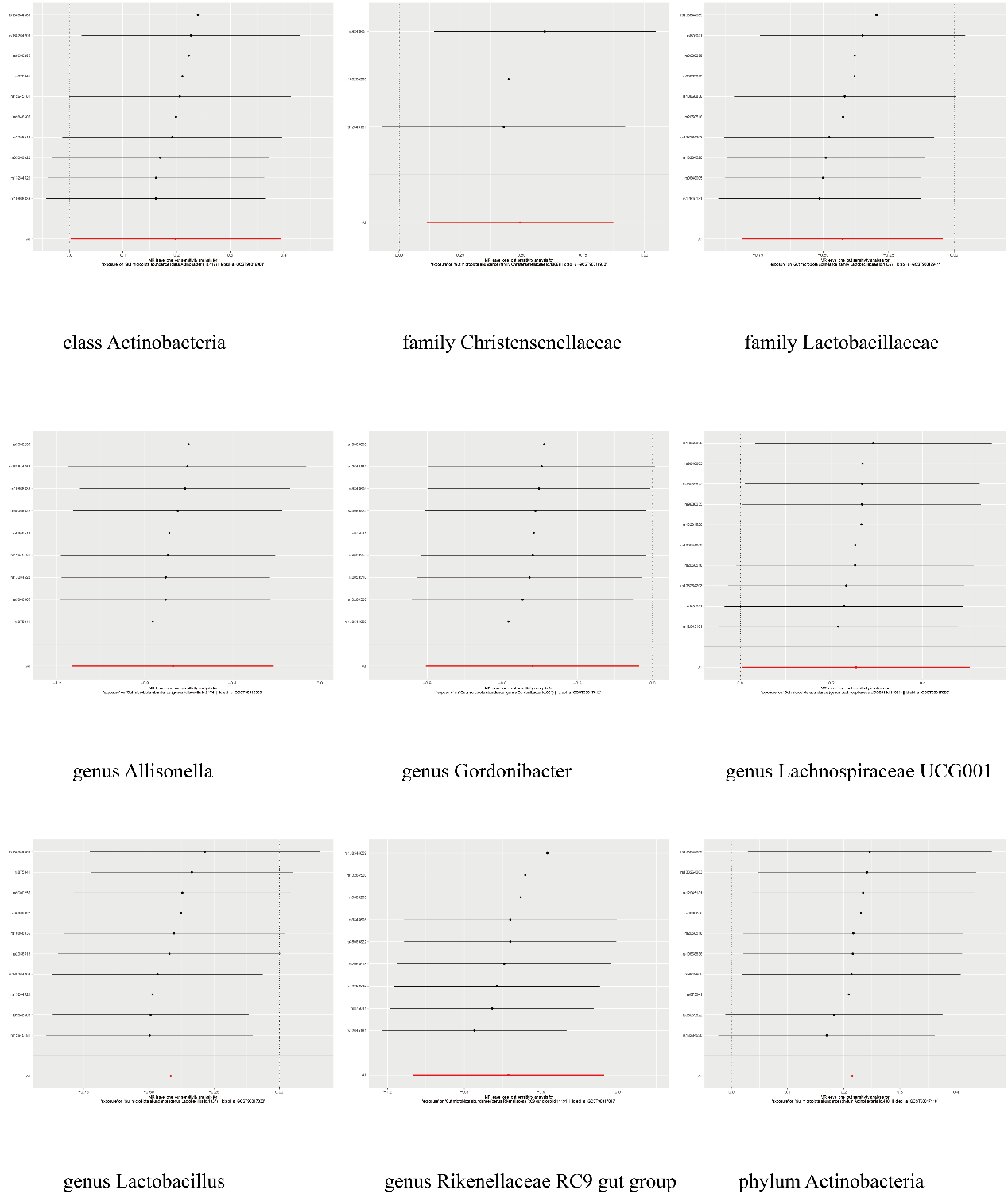


**Figure S4** Funnelplot, with nicotine dependence as exposure and gut microbiota abundance as result. (Only results with significant p-values in the IVW method are shown)


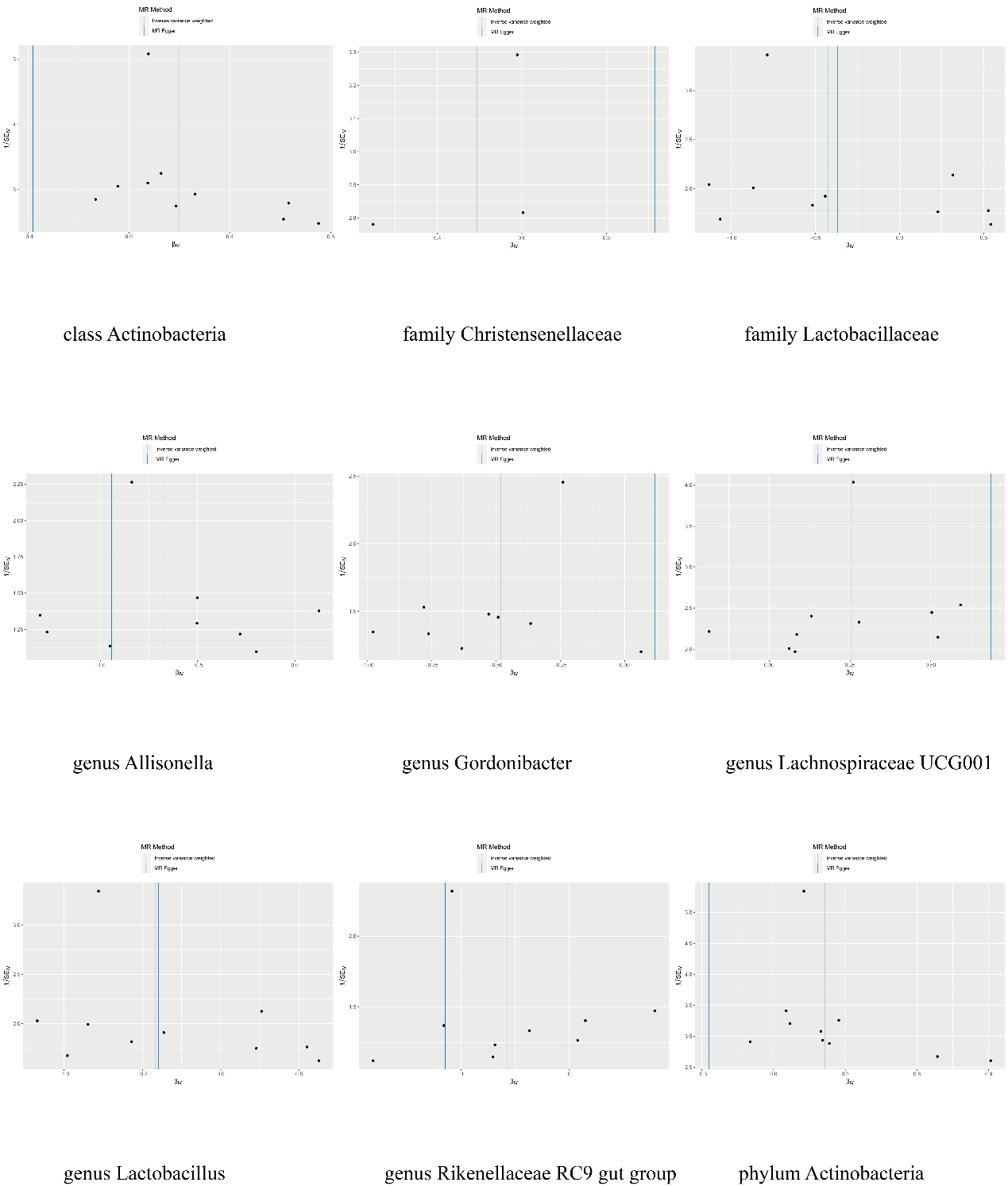


**Figure S5** Scatterplot of results of Mendelian randomization with gut microbiota abundance as exposure and nicotine dependence as outcome. (Only results with significant p-values in the IVW method are shown)


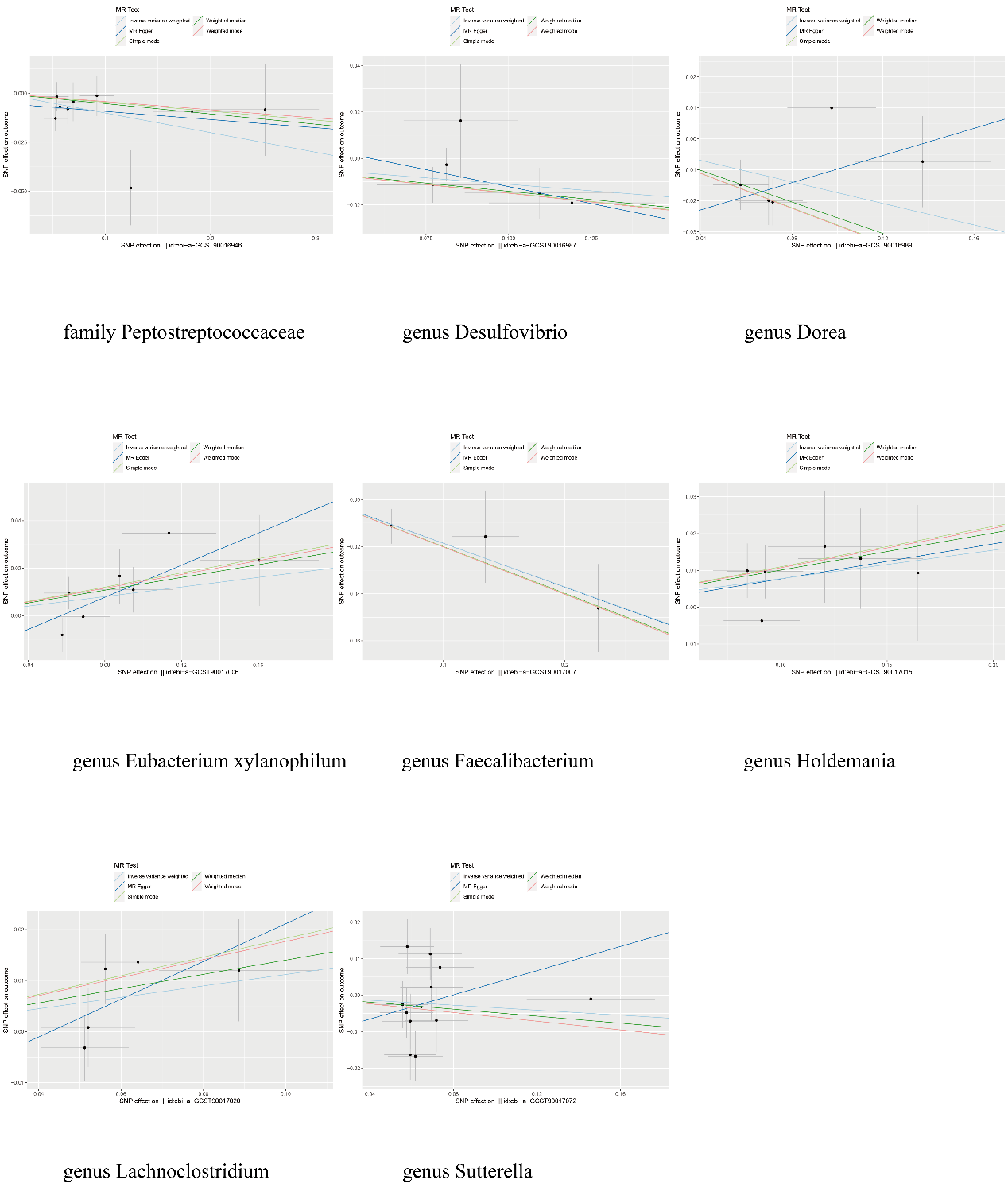


**Figure S6** Forest plot of single SNP analysis using Wald ratios, with gut microbiota abundance as exposure and nicotine dependence as outcome. (Only results with significant p-values in the IVW method are shown)


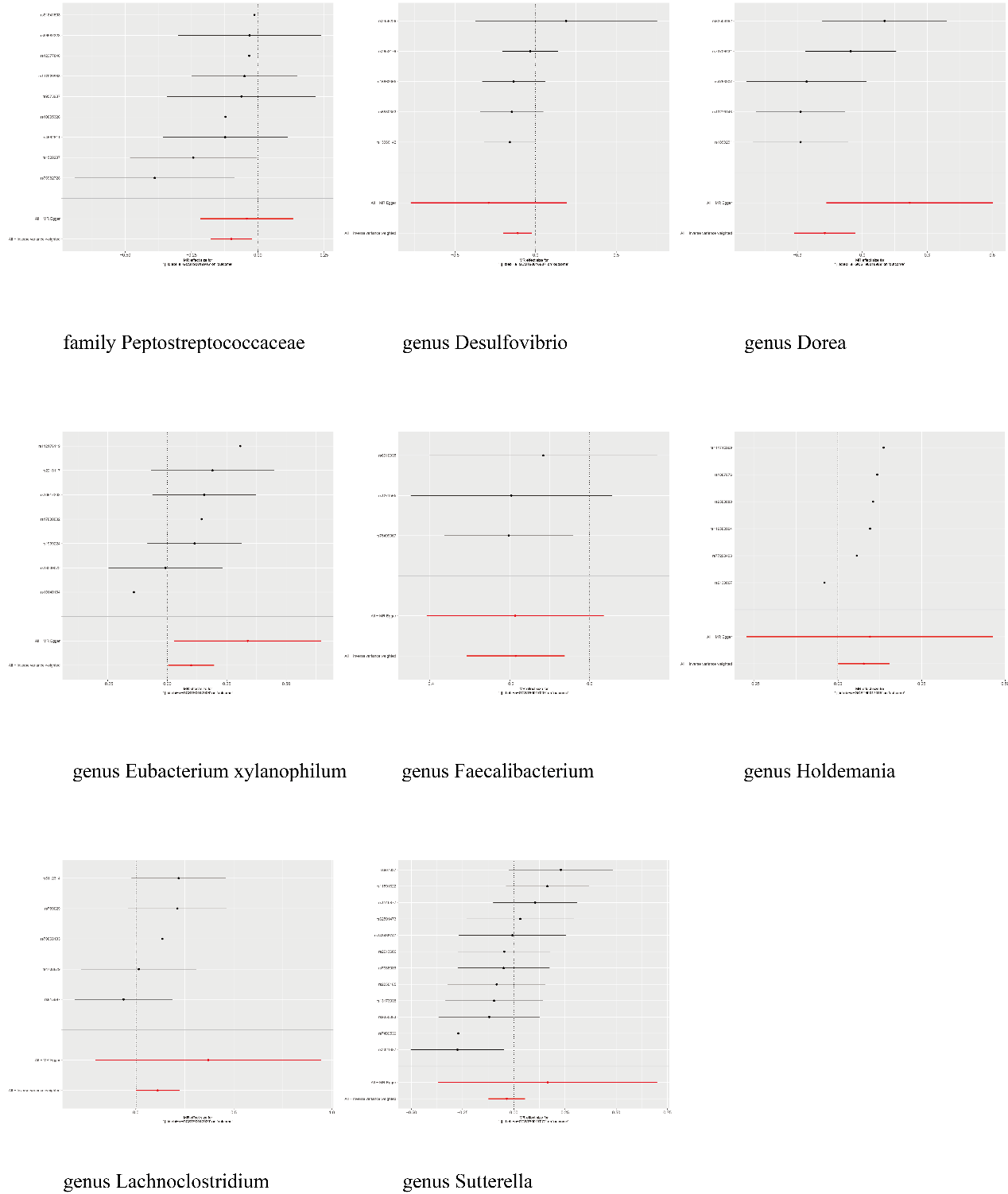


**Figure S7** Leave-one-out analysis plot, with gut microbiota abundance as exposure and nicotine dependence as outcome. (Only results with significant p-values in the IVW method are shown)


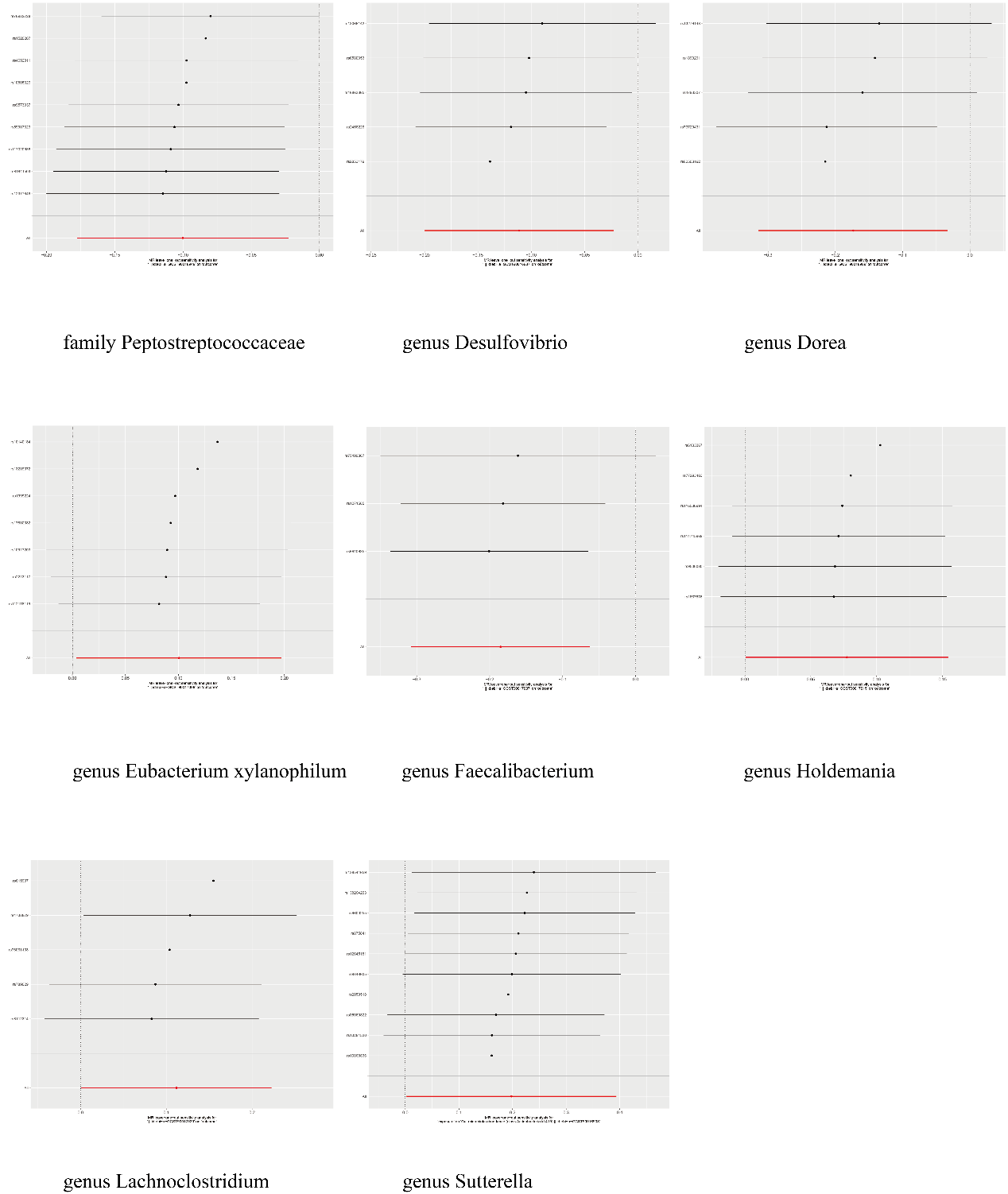


**Figure S8** Funnelplot, with gut microbiota abundance as exposure and nicotine dependence as outcome. (Only results with significant p-values in the IVW method are shown)


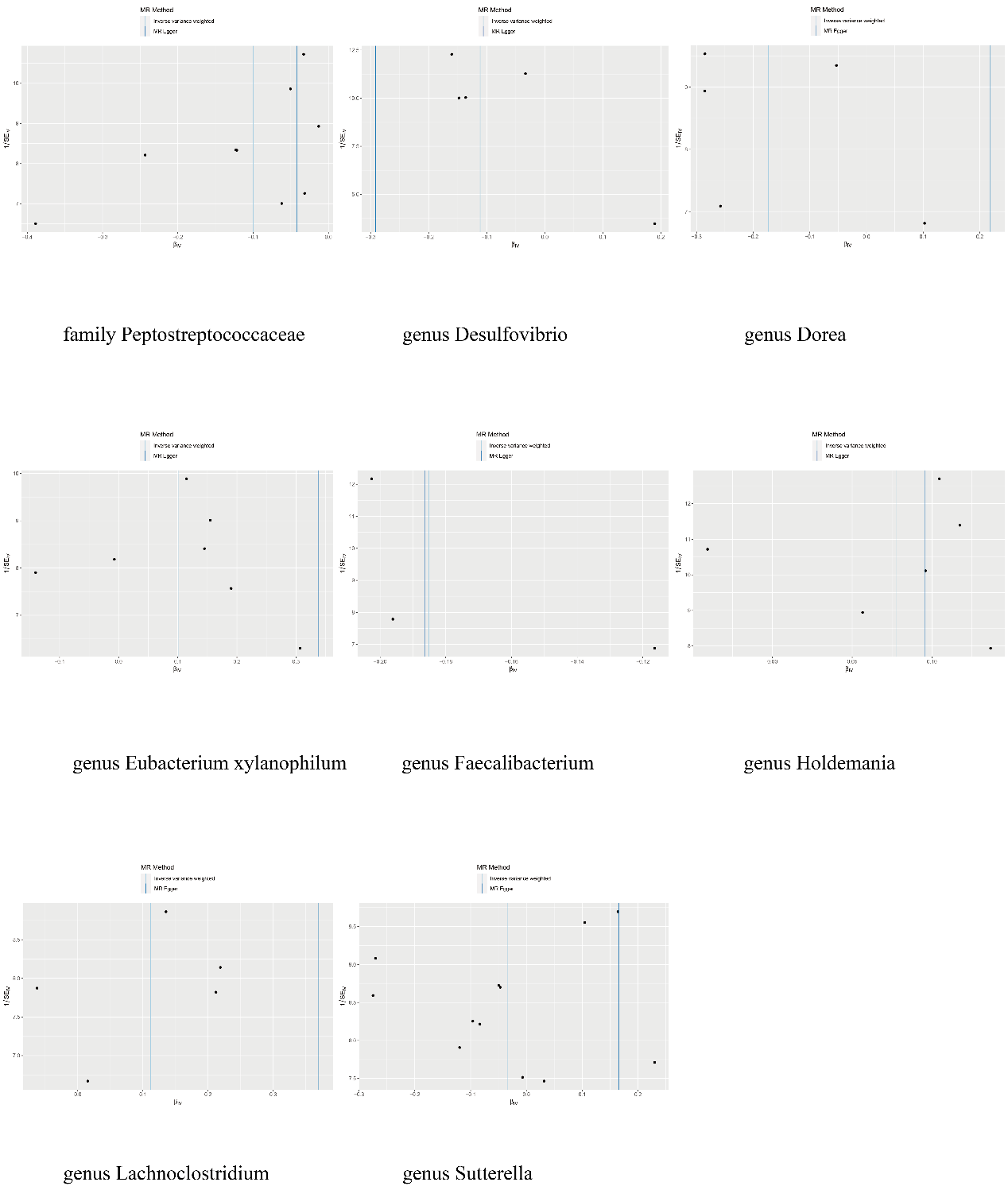

Supplement: Supplementary file 3 [file DataSheet_1.docx]
