# Supplementary material for: Association of nicotine dependence and gut microbiota: a bidirectional two-sample Mendelian randomization study
Source: Front Immunol. 2023 Nov 7;14:1244272. doi: 10.3389/fimmu.2023.1244272 (PMC10664251; doi:10.3389/fimmu.2023.1244272)
Supplement: Supplementary file 4 [file DataSheet_2.docx]

**Critical appraisal checklist for evaluating Mendelian randomisation studies**

| **Core Mendelian randomisation assumptions** | | |
| --- | --- | --- |
| Is there sufficient evidence that the genetic variants are robustly associated with the risk factor of interest? | We conducted extensive analysis and employed a large-scale genome-wide association study to identify genetic variations closely associated with the risk factors of interest. The p-value threshold used for our genome-wide association analysis was set at 5e-06, in addition to using an MAF threshold of 0.01. We also performed linkage disequilibrium analysis (R2 < 0.001, clustering distance of 10,000 kb). Finally, we calculated the F statistics for each SNP used as an instrumental variable, all of which exceeded 10. Please refer to Supplementary Tables S2 and S6 for further details. |  |
| Are the genetic variants associated with potential confounders? Do the authors present this relationship? | We employed various statistical methods, such as MR-Egger and MR-PRESSO, to assess and adjust for potential pleiotropic effects at the instrumental variable level. The results of MR-Egger and MR-PRESSO are presented in our supplementary tables S12, S13, S14, S15, and S16. Based on our results, we did not find evidence of pleiotropy at the instrumental variable level.  In addition, we employed multivariable Mendelian randomization to assess the potential confounding between other smoking phenotypes and nicotine dependence. Our results revealed that the instrumental variable phenotype for nicotine dependence is independent of instrumental variables for other smoking phenotypes, indicating a lower likelihood of potential confounding factors. |  |
| Is there any way for the genetic variants to affect the outcome through alternative pathways (horizontal pleiotropy)? Do the authors present alternative Mendelian randomisation approaches (such as MR Egger, median, and mode estimators, or use of “negative control” populations) to  investigate this more fully? | We employed various statistical methods, such as MR-Egger and MR-PRESSO, to assess and adjust for potential pleiotropic effects at the instrumental variable level. The results of MR-Egger and MR-PRESSO are presented in our supplementary tables S12, S13, S14, S15, and S16. Based on our results, we did not find evidence of pleiotropy at the instrumental variable level. We provide various Mendelian randomization methods for sensitivity analysis, including MR Egger, weighted median, simple mode, weighted mode, and mrpresso analysis. We also use a negative control group (smoking cessation phenotype). |  |
| **Methods reporting** | | |
| **All studies** | | |
| Are the effect and other alleles coded in the same direction for the exposure and outcome? | The effect and other alleles are coded in the same direction for both the exposure and outcome variables. |  |
| **Two sample studies** | | |
| Were the two samples drawn from the same population? | No |  |
| Were the two samples independent? | Yes |  |
| Was the analysis restricted to independent variants (that is, pruned of SNPs in linkage disequilibrium) or did the analysis allow for the correlation between variants? | Yes |  |
| **Data presentation** | | |
| Do the authors present the results as a genetic association, an instrumental variable estimate, or both? | We present the results as genetic association. |  |
| If they provide an instrumental variable estimate, do they compare it with the conventional observational estimate? | Not available. |  |
| Do the authors provide sensitivity analyses such as MR Egger, weighted median, and mode Mendelian randomisation, or use negative control populations? | We provide various Mendelian randomization methods for sensitivity analysis, including MR Egger, weighted median, simple mode, weighted mode, and mrpresso analysis. |  |
| Do the authors manually pick and choose which SNPs go into the instrument to tackle pleiotropy? If so, is the approach and justification clear? | No. |  |
| Do the authors provide the data that they used (especially for Mendelian randomisation analyses conducted at the summary level) in a supplement to allow researchers to reproduce their findings? | Yes, we provide the data they used, including Mendelian randomisation analyses conducted at the summary level, in a supplement to allow researchers to reproduce their findings. |  |
| **Interpretation** | | |
| If the Mendelian randomisation estimate is similar to the observational estimate and provides evidence in support of a causal effect, could it be due to weak instrument bias in a single study or confounding through, for example, horizontal pleiotropy? | The causal relationship between nicotine dependence and gut microbiota lacks previous observational estimates to compare with our Mendelian randomization results. However, in the results of our Mendelian randomization analysis, we did not find the existence of weak instrumental variable bias and horizontal pleiotropy of instrumental variables. |  |
| If the Mendelian randomisation estimate differs from the observational estimate and provides little evidence of a causal effect, could this be due to weak instrument bias when using two different samples or negative confounding due to pleiotropy? | The causal relationship between nicotine dependence and gut microbiota lacks previous observational estimates to compare with our Mendelian randomization results. However, in the results of our Mendelian randomization analysis, we did not find the existence of weak instrumental variable bias and horizontal pleiotropy of instrumental variables. |  |
| Mendelian randomisation provides estimates of the effects of the risk factor over a lifetime, and the numerical effect estimates may not be clinically meaningful. Will interventions at a specific age have the same sized effects? | Due to the fact that our Mendelian randomization analysis used summary data of nicotine-dependent phenotypes and gut microbiome from the whole-genome association study (GWAS) data, and individual GWAS data were not accessible, we are unable to determine the effectiveness of specific age-specific interventions. Further research is needed to identify the optimal timing and magnitude of these interventions. |  |
| Are the 95% confidence intervals of the Mendelian randomisation estimate sufficiently precise to identify the observational estimate and a clinically meaningful difference? | There is a lack of previous observational estimates regarding the causal relationship between nicotine dependence and gut microbiota to compare with our Mendelian randomization results. Therefore, this study is unable to identify any significant differences between the observed estimates and the clinically meaningful differences. |  |
| **Clinical implications** | |  |
| Do the results triangulate with other forms of evidence? Could a clinical trial be conducted to provide definitive evidence, as in the case of PCSK9 inhibitors? | There is a lack of previous observational estimates regarding the causal relationship between nicotine dependence and gut microbiota to compare with our Mendelian randomization results.  Our Mendelian randomization analysis was able to demonstrate the impact of nicotine dependence on gut microbiota abundance. Therefore, it may be possible to use certain microbiota that have a causal relationship with nicotine dependence as probiotics to intervene in nicotine dependence and provide effective means for its treatment. |  |
| If a randomised clinical trial is not feasible (such as in the case of alcohol consumption and risk of heart disease) or unlikely to be conducted in the short term (such as the case of lifestyle interventions to lower BMI and risk of heart disease), and there is existing evidence from multiple Mendelian randomisation studies and other robust study designs that converge on a similar result and show consistency of association, this information can be used to guide patient care; for example, advising weight loss to prevent heart disease or advising against moderate alcohol consumption to prevent cardiovascular disease | No, the analyzes derived from our Mendelian randomization studies cannot be used to guide patient care. |  |
